# Mass‐Produced and High‐Performance Nanowell Biosensor Fabricated via Semiconductor Manufacturing for Rapid and Accurate COVID‐19 Diagnosis in the Clinical Field

**DOI:** 10.1002/advs.202522897

**Published:** 2026-03-12

**Authors:** Yoo Min Park, Zahra Rezaei, Nam Ho Bae, Donggee Rho, Da‐Seul Kim, EunYoung Go, YoungTae Seo, Seok Jae Lee, Won Chan Seo, Luke P. Lee, HeaYeon Lee, Su Ryon Shin

**Affiliations:** ^1^ Center for Nano‐Bio Development National NanoFab Center (NNFC) Daejeon Republic of Korea; ^2^ Division of Engineering in Medicine Brigham and Women's Hospital Department of Medicine Harvard Medical School Cambridge MA USA; ^3^ Mara Nanotech INC. Hanmir Hall Yongdang Campus Pukyong National University Nam‐gu Republic of Korea; ^4^ Mara Nanotech New York INC. New York NY USA; ^5^ Department of Materials System Engineering Pukyong National University Nam‐gu Republic of Korea

**Keywords:** clinical trials, COVID‐19, electrochemical biosensors, nanowell array, point‐of‐care devices, SARS‐CoV‐2 antigen

## Abstract

Severe acute respiratory syndrome coronavirus‐2 (SARS‐CoV‐2) has underscored an urgent need for rapid, accurate, and accessible diagnostic tools to detect infections, facilitate timely quarantine, and inform therapeutic decisions. Fabricating nanostructures on biosensors can enhance biomolecule orientation, minimize steric hindrance, and reduce non‐specific binding, resulting in improved signal‐to‐noise ratios and high sensitivity, making them promising point‐of‐care diagnostic. However, clinical translation remains limited by challenges in scalable and reproducible fabrication and insufficient diagnostic validation. Here, we introduce a nanowell biosensor (NW‐Biosen) fabricated via semiconductor manufacturing, enabling scalable production and yields >1000 electrodes with reproducible properties. NW‐Biosen detects SARS‐CoV‐2 antigens from patient nasal swabs within ∼10 min, with high sensitivity and minimal interference from other coronavirus recombinant proteins, respiratory pathogens, bacteria, and environmental substances, while maintaining consistent reproducibility with different batches. We validated NW‐Biosen through two independent clinical trials involving 249 retrospective and 243 prospective patient samples. In prospective cohort, NW‐Biosen achieved 93.02% sensitivity, 98.73% specificity, and a Cohen's kappa of 0.927, indicating near‐perfect agreement with RT‐PCR and superior sensitivity compared to commercially available colorimetric kits. Thus, NW‐Biosen enables rapid, highly sensitive, reproducible, and cost‐effective at‐home detection, with real‐time data transmission to public health authorities via mobile app integrated with a miniature potentiostat.

## Graphical abstract

A semiconductor‐fabricated nanowell biosensor enables rapid, scalable, and highly reproducible detection of SARS‐CoV‐2 antigens from nasal swabs within ∼10 minutes. Clinical validation in 249 retrospective and 243 prospective patient samples demonstrates high sensitivity and specificity, minimal cross‐reactivity, and robust batch‐to‐batch reproducibility, supporting low‐cost at‐home testing with smartphone‐integrated readout for real‐time reporting.

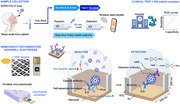

## 1 Introduction

The 2019 Novel Coronavirus (COVID‐19) pandemic, caused by severe acute respiratory syndrome coronavirus 2 (SARS‐CoV‐2), has created significant global health challenges, demanding the development of rapid, accurate, and reliable diagnostic tools for effective disease management [1]. The current gold standard for SARS‐CoV‐2 detection is reverse transcription‐polymerase chain reaction (RT‐PCR), which offers high analytical sensitivity and specificity. However, RT‐PCR's applications are limited by the need for centralized laboratories, specialized equipment, and skilled personnel, resulting in long turnaround times and logistical challenges for point‐of‐care (POC) use [2, 3]. To overcome these limitations, various alternative diagnostic platforms have been introduced, including reverse transcription‐loop mediated isothermal amplification (RT‐LAMP), clustered regularly interspaced short palindromic repeats (CRISPR)‐based diagnostics, and lateral flow immunoassays. While these methods improve accessibility and turnaround time, they often require pre‐amplification, suffer from variable sensitivity in low viral load scenarios, or lack the robustness necessary for clinical implementation at the POC level [4, 5].

Among these alternatives, electrochemical biosensors have gained substantial attention due to a few inherent advantages, such as rapid signal acquisition, high sensitivity and specificity, and compatibility with portable systems through miniaturization, making them particularly attractive for decentralized diagnostics [6, 7]. However, electrochemical biosensors for SARS‐CoV‐2 detection are primarily based on antibody‐antigen detection, which, while faster and more accessible, is generally less sensitive than PCR‐based methods, particularly during early infection stages when antibody or antigen levels may be below detection thresholds. To overcome these limitations, the incorporation of nanostructure, particularly nanowell architecture, on biosensors can make them capable of detecting extremely low antigen concentrations. Nanowell architectures create confined reaction areas that optimize biomolecular orientation, minimize steric hindrance, and significantly reduce non‐specific binding, resulting in improved signal‐to‐noise ratios and enhanced analytical performance, even at low analyte concentrations in complex biological matrices [8, 9]. Furthermore, the ability to precisely control nanowell geometry (depth, diameter, and pitch) enables accurate spatial confinement of analytes, thereby improving selectivity [9]. Our previous studies have reported up to 150‐fold increases in sensitivity using nanowell designs compared to flat electrodes [8]. Despite these advantages, the transition of nanostructured biosensors from proof‐of‐concept research to validated POC diagnostics in clinical settings has been hindered by several challenges, including high manufacturing costs, scalability, and reproducibility of nanostructured biosensors, as well as their subsequent impact on biosensor function in clinical validation. Traditional methods such as electron‐beam lithography or focused ion beam milling are low‐throughput and costly, and often lack the reproducibility needed for clinical‐grade sensor arrays [10, 11]. More critically, as production scales up, maintaining consistent nanowell geometry and surface chemistry becomes increasingly difficult, which can compromise sensitivity, specificity, and overall device reproducibility, which are all key metrics for regulatory approval and clinical deployment [9].

To overcome manufacturing barriers, we employed a semiconductor manufacturing process using krypton‐fluoride (KrF) scanner lithography. This technique, widely adopted in the semiconductor industry for high‐resolution patterning, offers nanoscale precision (typically ∼200 nm) within large wafer areas, enabling the batch fabrication of thousands of sensors with minimal variation [12]. The KrF scanner process, which allows for photoresist patterning on polymer or glass substrates, enables flexible integration with various biosensing modalities [9, 13]. However, implementing semiconductor lithography in biosensor production introduces several challenges. For example, ensuring good compatibility between photoresists and biological surfaces, and the accurate alignment of multistep layers for nanowell depth control are non‐trivial engineering hurdles [10, 14]. Furthermore, minimizing sensor‐to‐sensor variation within the wafer is essential for maintaining reproducibility. While sensitivity is primarily influenced by the quality and uniformity of gold deposition, selectivity can be enhanced through precise bioreceptor alignment with nanowalls or by choosing appropriate bioreceptor types.

**SCHEME 1:**
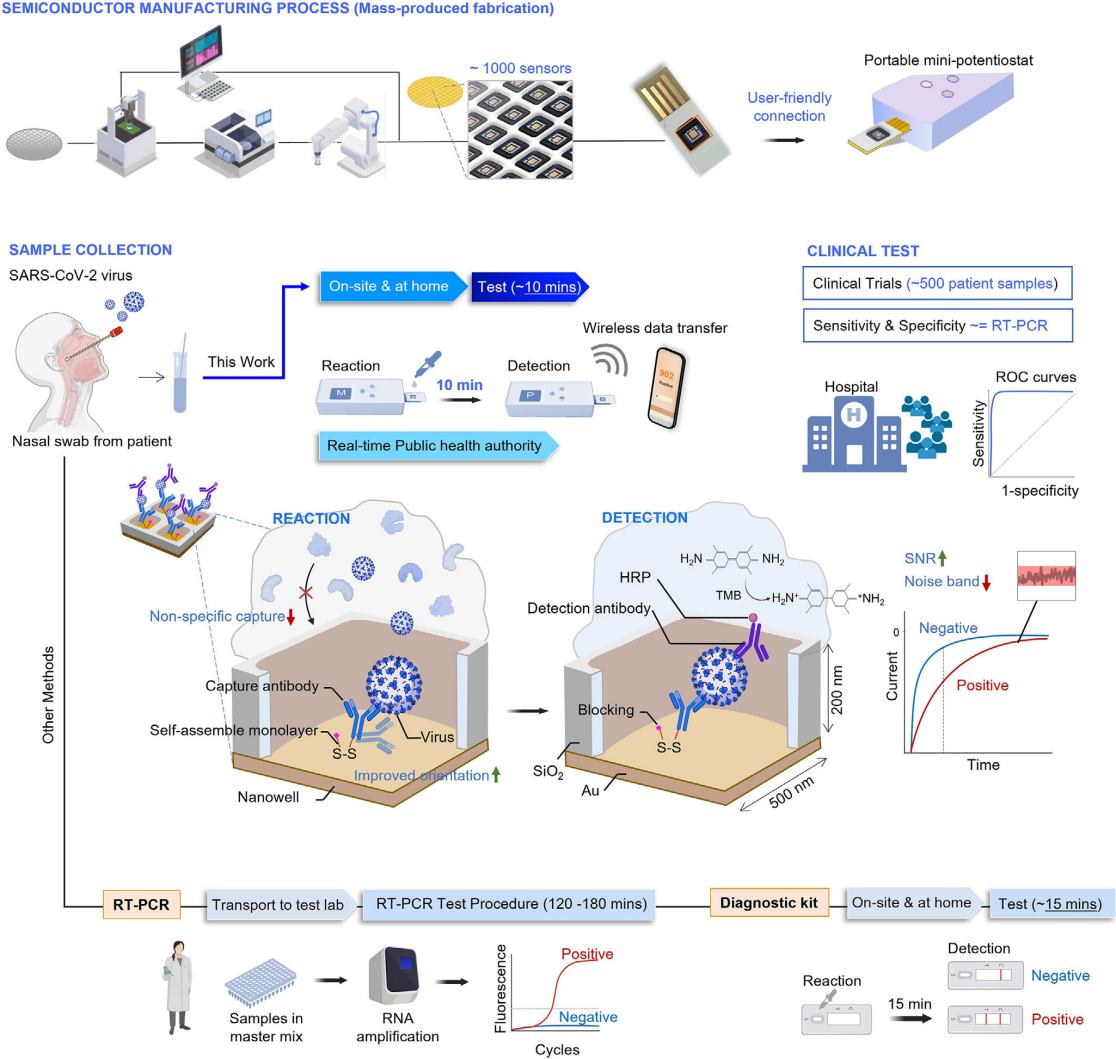
Schematic representation of SARS‐CoV‐2 detection approaches using the NW‐Biosensing POC kit compared to conventional diagnostic systems. This figure compares conventional diagnostics, such as RT‐PCR, which requires centralized laboratory testing and takes 120–180 min, and commercial colorimetric POC kits, which provide qualitative results in ∼15 min, with a proposed portable electrochemical biosensing kit. The mass‐produced NW‐Biosen was fabricated using a semiconductor manufacturing process, showing rapid (∼10 min) antigen detection with excellent sensitivity and selectivity achieved by the advantages of adding nanowell architecture, and a portable potentiostat for real‐time wireless data transmission to smartphones. The NW‐Biosensing POC kit has undergone clinical trials with ∼500 patient samples, demonstrating its potential for decentralized testing environments and rapid communication with healthcare authorities to enable timely intervention. SNR: signal‐to‐noise ratio.

In this study, we developed a scalable, portable, reproducible, and high‐performance nanowell array‐based EC biosensor for the rapid detection of the SARS‐CoV‐2 nucleocapsid protein antigen from anterior nasal swabs and clinical samples (Scheme 1). The electrode with a nanowell array was fabricated using a semiconductor manufacturing process, enabling the precise patterning of nanowells on large surface areas while ensuring high reproducibility and uniformity, which are critical features for clinical‐grade diagnostics. The reproducibility, structural integrity, and consistency of the nanowell array on the electrodes were characterized using a scanning electron microscope (SEM), while the electrical and electrochemical properties were evaluated through resistance measurements, cyclic voltammetry (CV), square wave voltammetry (SWV), and electrochemical impedance spectroscopy (EIS). The surface of nanowell electrodes were subsequently functionalized by antibodies for immunoassay‐based detection with horseradish peroxidase (HRP)‐conjugated antigens to amplify the electrical signal. Chronoamperometry (CA) was employed using a miniaturized potentiostat that facilitates rapid readout and real‐time data transmission wirelessly to smartphones and public healthcare authorities in alignment with POC testing needs. We further evaluated selectivity against another 2 coronavirus recombinant proteins, 5 different SARS‐CoV‐2 variants, 15 other respiratory pathogens, 8 bacterial species, and 13 environmental substances, all of which showed minimal interference.

The nanowell biosensor's (NW‐Biosen) analytical performance was validated through two independent clinical studies conducted at separate hospitals, utilizing both retrospective and prospective samples obtained from nasal swabs. Importantly, we directly measured patient samples on‐site using a miniaturized potentiostat and sent the obtained data wirelessly to public healthcare authorities, confirming the real‐world clinical applicability of the device. This work demonstrates how semiconductor manufacturing can be used as a mass‐production method for fabricating NW‐Biosen with high sensitivity, selectivity, and reproducibility, while maintaining a rapid and robust sensing performance in diverse clinical settings and sample conditions through the usage of nanowell architectures. By addressing key translational barriers, the NW‐Biosen platform advances nanostructured biosensing toward widespread clinical and POC deployment compared with conventional SARS‐CoV‐2 detection methods, such as RT‐PCR and commercial colorimetric POC diagnostic kits.

## 2 Results

### 2.1 Mass Production of Nanowell Electrodes via Semiconductor Manufacturing Process and Reproducibility Assessment

**FIGURE 1:**
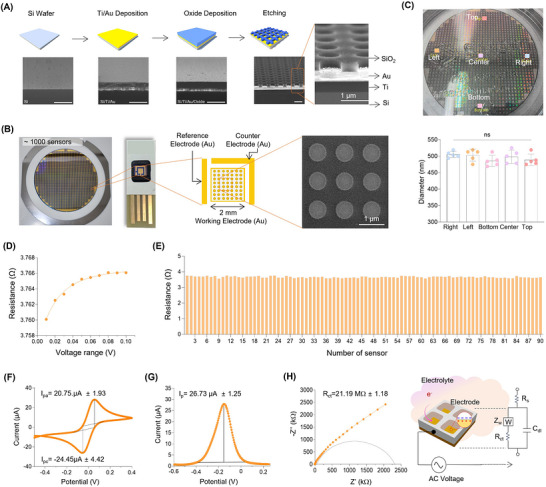
Mass production of nanowell electrodes and reproducibility evaluation. (A) Schematic illustration of the nanowell electrode fabrication process via deposition, photolithography, etching, along with SEM images depicting each step. Ti (20 nm), Au (200 nm), and SiO_2_ (200 nm) layers were sequentially deposited. (B) Manufactured nanowell electrode with a SEM image, showing nanowell sensors on an 8‐inch wafer and a USB‐type PCB package. Approximately 1000 sensors were produced on a single wafer. (C) Consistency evaluation of nanowell diameter in different positions on the semiconductor wafer (n = 5 at 5 different areas, total n = 25). Differences were not statistically significant (ns). (D) Resistance versus voltage plot for a representative sensor, indicating stable performance (R_avg_ = 3.665± 0.049, n = 90). (E) Resistance values of 90 sensors, demonstrating consistent fabrication quality. Electrochemical signals of nanowell electrodes with (F) CV (scan rate: 50 mV/s, potential range of −0.6 to 0.6 V), (G) SWV (increment: 5 mV, amplitude: 25 mV, frequency: 10 Hz), and (H) EIS (16 Hz to 0.01 Hz, amplitude: 10 mV) in 5 mm ferricyanide/KCl with their equivalent circuit model were used to characterize the bare electrode electrochemically (n = 11). (Data = mean ± SD).

To enable large‐scale fabrication of reproducible and high‐performance nanowell electrodes, nanowell array with diameters from 230 to 500 nm were fabricated on the working electrode through a semiconductor manufacturing process. The approximate number of nanowells with diameters of 230, 250, and 500 nm were ∼18.9 million, ∼16 million, and ∼4 million, respectively. This process involved a two‐step, high‐throughput KrF scanner photolithography technique, including thin‐film metal deposition followed by reactive dry ion etching to define uniform nanowell structures. The successful deposition of each layer was verified using SEM analysis, and final cross‐sectional SEM images confirmed that the nanowell structures were constructed as designed. As shown in Figure 1A, the semiconductor‐based fabrication process involved the sequential deposition of titanium (Ti, 20 nm), gold (Au, 200 nm), and silicon dioxide (SiO_2_, 200 nm) layers onto an oxidized 8‐inch silicon wafer, followed by nanowells 500 nm in diameter. A titanium adhesion layer ensured robust gold deposition onto silicon wafers [15], with an Au layer on top, chosen for its superior conductivity, stability, and biocompatibility. Silicon dioxide served as an insulating layer, selectively exposing regions for bioreceptor immobilization and further improving sensitivity and signal accuracy [16]. As shown in Figure S1, nanowells with 230 and 250 nm diameters also illustrate the ability to fabricate well‐defined, uniform nanowell geometries with different sizes and surface topographies, confirming the precision and reproducibility of the nano‐fabrication process. Finally, through the optimized semiconductor manufacturing process, nanowell electrodes were fabricated with a dot grid pattern featuring a pitch and diameter of 500 nm for over 1000 electrodes on an 8‐inch wafer. To achieve the on‐chip sensing system, we deposited reference (RE) and counter (CE) electrodes using gold next to the nanowell arrayed working electrode (WE). Au was selected as the reference electrode material due to its chemical inertness, biocompatibility, and compatibility with standard microfabrication processes. Unlike Ag/AgCl, which can leach Ag^+^ ions and requires chloride‐rich conditions for stable potential, Au provides stable pseudo‐reference behavior with diverse electrolytes and biological media. Several prior studies have successfully integrated Au as a pseudo‐reference electrode in miniaturized biological electrochemical sensing platforms [17, 18, 19]. The obtained electrode was integrated onto a USB‐type printed circuit board (PCB), designed for user‐friendly handling (Figure 1B). This compact format confined the solution to a defined area while enabling seamless integration with portable, miniatured potentiostats. To confirm the uniformity of the fabricated nanowells on the entire wafer, nanowell diameters were measured from five randomly selected electrodes located at the top (488.8 ± 17.0 nm), center (498.0 ± 17.8 nm), bottom (485.8 ± 16.8 nm), left (502.8 ± 18.6 nm), and right (505.0 ± 10.0 nm) regions of the wafer. Although Figure 1C shows a slightly lower well diameter for the electrodes at the bottom region of the wafer compared with the top region, there were no significant differences. This level of variation is consistent with expected process‐related fluctuations in wafer‐scale nanofabrication and still demonstrates high uniformity, validating the scalability and reproducibility of the fabrication process.

In our previous studies, we investigated the improved electrochemical sensing performance of 500 nm‐diameter nanowell electrodes compared with flat sensors [20]. After demonstrating the feasibility of fabricating nanowell electrodes of varying sizes and uniformity via a semiconductor manufacturing process at large scale, a nanowell electrode with a 500 nm diameter was selected because it yielded a more reproducible and stable electrochemical signal, enabling evaluation of biosensor performance for mass production and commercialization. The electrochemical stability and fabrication uniformity of the nanowell sensor array were characterized through resistance measurements (Figure S2). In Figure 1D, resistance values were plotted against an applied voltage range from 0.01 to 0.1 V. The resistance remained stable at lower voltages, exhibiting an approximately linear response consistent with near‐ohmic behavior. At higher applied voltages, a slight deviation from ideal linearity was observed, with a tendency toward resistance saturation. This behavior can be attributed to contact resistance contributions and local charge transport limitations under increased electrical bias, and is consistent with established electrochemical transport principles [21]. The resistances of 90 individual nanowell electrodes are shown in Figure 1E, with values tightly distributed around 3.8 ± 0.1 Ω. Minimal sensor‐to‐sensor variation was observed, validating the platform's batch reproducibility, which is critical for applications in both market and clinical fields. Together, these results demonstrate that the nanowell sensors exhibit electrically stable and ohmic characteristics, with high fabrication precision, which lays a reliable foundation for subsequent electrochemical analyses using CV, SWV, and EIS with ferricyanide/potassium chloride (KCl) electrolyte. Figure 1F shows the CV results with clearly defined and symmetric oxidation (20.75 ± 1.93 µA) and reduction (−24.45 ± 4.42 µA) peaks, demonstrating consistent electrochemical behavior. The peak‐to‐peak voltage difference was approximately 0.1 V, which indicates rapid electron transfer between the working and counter electrodes. This narrow separation reflects efficient and reversible redox activity, and supports the high quality of the sensor surface and electrode design [21]. In Figure 1G, SWV data exhibited sharp, repeated current peaks without noticeable noise or irregularities (26.73 ± 1.25 µA). The consistency between electrodes confirms the uniformity of the fabrication process and the stability of the functional layer, which are essential for reliable sensing performance [21]. Additionally, EIS was used to evaluate the interfacial properties of the electrodes, and the data is shown in Figure 1H. The impedance signal was fitted using an equivalent circuit model containing components for solution resistance (R_s_), charge transfer resistance (R_ct_), double‐layer capacitance (C_dl_), and diffusion‐related impedance (Z_w_). The charge transfer resistance was approximately 21.19 ± 1.18 MΩ. Characterization was performed using SEM imaging and electrochemical techniques, including CV, SWV, and EIS, and the results confirmed the uniformity of the nanowell geometry and the reproducibility of charge transfer behavior between batches. Minimal sensor‐to‐sensor variation (standard deviation <0.1 Ω) was shown, validating the platform's batch reproducibility. The absence of pronounced nonlinear deviations in the EIS response indicates stable electrical contact and uniform interfacial properties of devices. Despite relatively high R_ct_ values, narrow inter‐sensor variability affirmed consistent electron transfer and robust redox activity.

### 2.2 Biosensing Performance of Nanowell Electrodes

**FIGURE 2:**
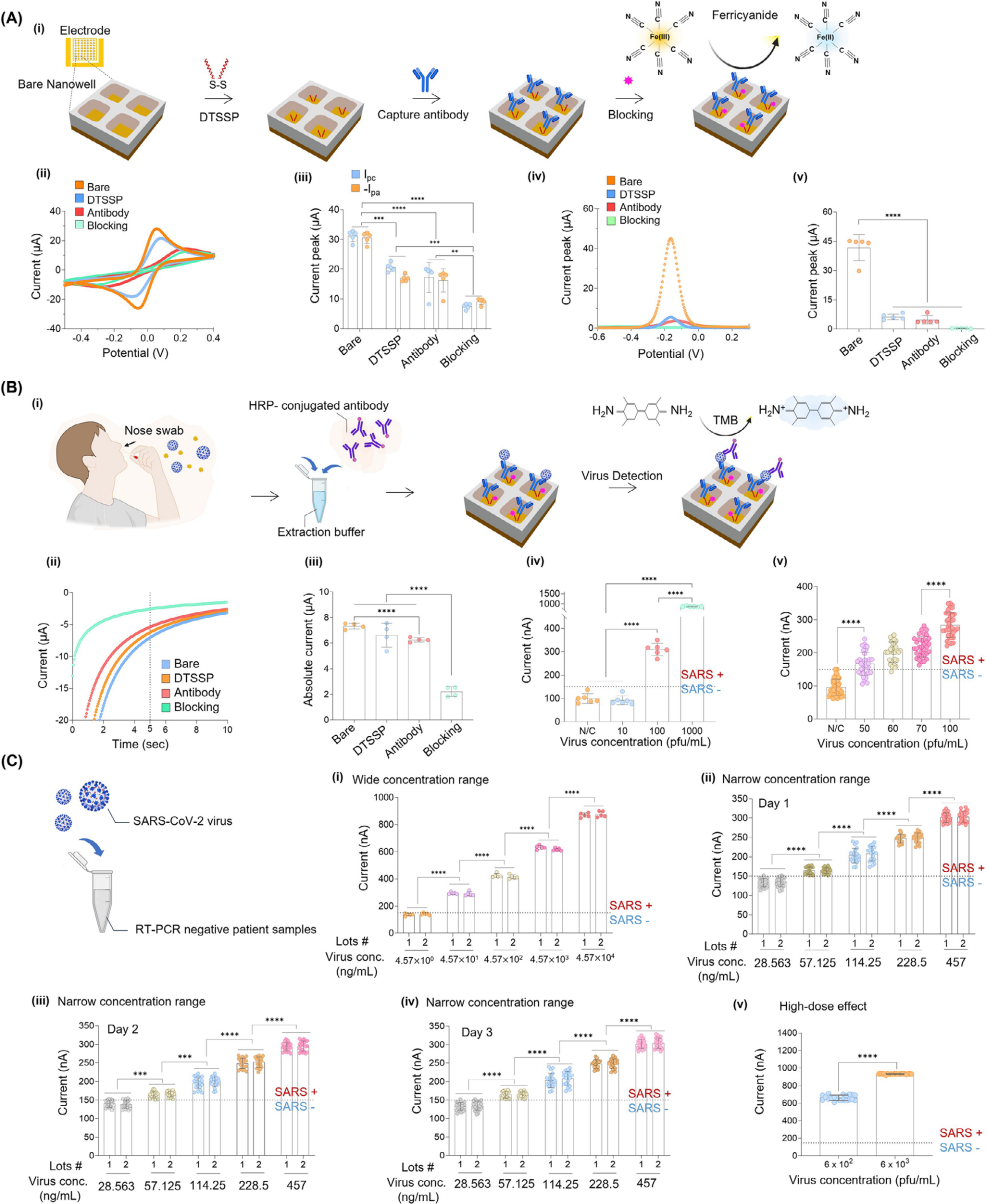
Electrode functionalization, virus detection, and reproducibility of NW‐Biosen using patient swab samples. (A‐i) Schematic of functionalization and preparation process of the NW‐Biosen, (A‐ii) CV curves obtained at each step: bare, DTSSP, antibody, and blocking, and (A‐iii) the corresponding current peaks (scan rate:50 mV/s, potential range of −0.6 to 0.6 V, interval: 1 mV). (A‐iv) SWV curves (increment: 5 mV, amplitude: 25 mV, frequency: 10 Hz) show a progressive decrease in (A‐v) current peaks, confirming successful functionalization (n = 5). Measurements were performed in 5 mm ferricyanide/KCl electrolyte using benchtop CHI 660E potentiostat. (B‐i) Schematic of sample preparation and detection, and (B‐ii) CA curves (initial potential: 0 V, low potential: −50 mV, pulse width: 10 s, interval: 0.05 s) and (B‐iii) the changes in the peak with corresponding values (n = 4). (B‐iv) Concentration threshold for a positive response (n = 6 for NC,10, 100, 1000 pfu/mL samples). (B‐v) Concentration threshold for a positive response in a narrow concentration range (n = 60 for NC sample and _n_ = 40 for 50, 60, 70 pfu/mL samples). Measurements in (B‐iv,v) were performed in TMB electrolyte using a mini‐potentiostat. Spiked samples were prepared by adding known concentrations of virus into commercial extraction buffer. (C‐i) Sensor response in a wide range of concentration levels using patient swab samples from two production lots of NW‐Biosen, demonstrating signal consistency (n = 5). (C‐ii–iv) Reproducibility validation on three separate days (Day 1, Day 2, Day 3) with narrower concentration range, showing consistent SARS‐CoV‐2 detection between sensor batches. Spiked samples were prepared by adding known concentrations of virus into negative patient swab samples (n = 20). (C‐v) Evaluation of high‐dose effects comparing responses at 6 × 10^2^ (low concentration) and 6 × 10^3^ pfu/mL (high concentration) (n = 20). Spiked samples were prepared by adding known concentrations of virus into commercial extraction buffer. (Data = mean ± SD, **p‐value< 0.05, ***p‐value<0.001, ****p‐value < 0.0001; Unpaired two‐tailed *t*‐test for two groups. One‐way ANOVA with Tukey for ≥3 groups. Adjacent comparisons only; ns results not shown).

Before optimizing the functionalization process, we first validated the detection of SARS‐CoV‐2 with various concentrations by targeting three specific genes (RdRp, E, and N) in the viral RNA. To do this, we used RT‐PCR to measure the cycle threshold (C_t_) values of each gene, which confirms the actual viral load in each sample. There is a clear inverse relationship between C_t_ values and viral concentration, as shown in Table S1, confirming that the RT‐PCR assay is both sensitive and accurate, even at low viral loads (1.56 × 10^1^ pfu/mL). The consistent detection of all three genes with different concentrations further supports the reliability of these RT‐PCR results. The results were then used as a standard method to validate our biosensor's performance. To ensure reliable biosensor performance, proper functionalization of the electrode surface was essential. The biosensor functionalization steps are illustrated in Figure 2A‐i. A monoclonal antibody with high target specificity was selected as the biorecognition element and was covalently immobilized onto the electrode using 3,3'‐dithiobis (sulfosuccinimidyl propionate (DTSSP) as the surface linker via Au–Sulfur binding. DTSSP is a hydrophilic, homo‐bifunctional crosslinker that forms stable self‐assembled monolayers on gold. DTSSP's sulfo‐NHS groups enable direct covalent bonding with primary amines on proteins, simplifying the protocol by eliminating 1‐Ethyl‐3‐(3‐dimethylaminopropyl)carbodiimide/N‐Hydroxysuccinimide (EDC/NHS) activation steps. Its short molecular length and hydrophilic properties reduce nonspecific adsorption while preserving electron transfer efficiency [22]. This addition streamlined the functionalization process enhances reproducibility and sensor performance.

After antibody immobilization, a blocking buffer (1% bovine serum albumin (BSA) and 0.1% Triton X‐100 in phosphate‐buffered saline (PBS)) was applied to passivate unreacted sites and suppress nonspecific binding. CV and SWV measurements in a ferricyanide/KCl electrolyte confirmed successful surface modification and background signal reduction. As demonstrated by the representative CV curves (Figure 2A‐ii) and the corresponding peak currents at *Ipc* and *Ipa* (Figure 2A‐iii), immobilization of each functional layer resulted in a significant decrease and shift of the redox peak currents. SWV consistently offers higher sensitivity and better suppression of background current; its implementation also demonstrated a stepwise signal reduction in the current peaks upon layer immobilization, with a more distinct decrease compared to CV (Figure 2A‐iv,v). Moreover, the reported parameters were obtained through an empirical assay development process, which focused on key factors affecting sensor performance, including plasma surface treatment (no treatment, N_2_, or O_2_ plasma), blocking buffer formulation, incubation times for DTSSP and antibodies, and 3,3′,5,5′‐tetramethylbenzidine (TMB) reaction time (Figure S3). Among these, O_2_ plasma treatment significantly improved signal clarity and selectivity between positive and negative samples. Plasma treatment, particularly O_2_ plasma, rapidly removes organic contaminants and enhances the cleanliness and hydrophilicity of the Au surface, thereby promoting the formation of well‐ordered SAMs, which is favorable for uniform antibody immobilization. This improvement is consistent with prior studies showing that O_2_ plasma cleaning of Au substrates removes contaminants and introduces oxygen‐containing groups that promote well‐ordered self‐assembled monolayer formation, thereby enhancing antibody immobilization and reducing nonspecific binding [23].

To develop a POC system, CA was employed to detect electron transfer events from the enzymatic reaction within a few seconds. CA was selected for its speed, high sensitivity, and straightforward data interpretation [24]. The reaction used TMB as the redox substrate. To amplify the electrochemical signal after binding the virus, an HRP‐conjugated antibody was employed. This enzymatic turnover acts as a natural signal amplification, which is essential for detecting the low antigen levels typically present in clinical samples. Prior to introducing the HRP‐labeled detection antibody and TMB, each surface‐assembly step deposited a largely nonconductive organic layer. After incubation with the HRP‐conjugated antibody and addition of TMB, the transduction shifted to enzyme‐amplified CA. In this reaction, HRP catalyzes TMB oxidation, and the electroactive product is rapidly cycled at the electrode, making the measured current proportional to the amount of bound HRP and, therefore, the antigen concentration (greater antigen equals higher current) [25]. In addition, TMB is electroactive and directly measurable, which yields stronger and faster responses; Figure 2B‐i provides a schematic representation of this process, where the target sample, obtained from a nose swab, is conjugated with the label through an HRP‐conjugated monoclonal antibody and is then introduced to the electrode. The functionalization process was further validated using TMB measurements. Figure 2B‐ii illustrates the stepwise decrease in the CA signal slope at each functionalization step up to the blocking step, confirming the process's success. The absolute current value at 5 s was obtained from several sensors (Figure 2B‐iii), and this decrease was further validated within all steps, indicating a similar trend compared to the SWV or CV signals while monitoring sensor preparation. Once functionalization was confirmed, a qualitative test was performed using a mini‐potentiostat with deactivated SARS‐CoV‐2 virus spiked into the commercial extraction buffer to determine the biosensor's lower limit of detection (LOD). The measurements were compared to results obtained using RT‐PCR. Serial dilution tests were conducted at virus titers of 1.0 × 10^3^, 1.0 × 10^2^, and 1.0 × 10^1^ pfu/mL using commercial extraction buffer. The results demonstrated that titers of 1.0 × 10^3^ and 1.0 × 10^2^ consistently yielded positive results, while 1.0 × 10^1^ titers were consistently negative, highlighting the kit's ability to distinguish between high, medium, and low viral concentrations (Figure 2B‐iv). Additional experiments near the LOD were conducted at virus titers of 1 × 10^2^, 7 × 10, 6 × 10, and 5 × 10 pfu/mL for 40 replicates. Positive detection rates were as follows: at 1 × 10^2^ and 7 × 10, 100% of replicates tested positive; at 6 × 10, 98% of replicates tested positive; and at 5 × 10, 68% of replicates tested positive (Figure 2B‐v). These findings demonstrate the NW‐Biosen's high sensitivity, reliably detecting viral concentrations at or above 6 × 10 pfu/mL. The LOD was determined to be 6 × 10 pfu/mL, with a positive detection rate of 95% or higher within 20 repeated tests. In the next step, the reproducibility and stability of the sensor response were evaluated by testing spiked virus in RT‐PCR–confirmed negative patient samples (Table S2). The evaluation was first performed in a wide range of viral concentrations (from 45.7 × 10^0^ to 45.7 × 10^4^ ng/mL) in Figure 2C‐i, and subsequently in a narrower concentration range over three separate days using individual electrodes from two different fabrication lots (Figure 2C‐ii–iv).

The current cut‐off value for the NW‐Biosen was also determined to be able to be used to distinguish between positive samples (those containing the target analyte) and negative samples. A total of 30 samples from two different product lots were measured. The obtained value from CA measurements using a mini‐potentiostat was 96.2 ± 24.2 nA. Then the cut‐off threshold was calculated by adding 1.65 times the standard deviation to the mean, resulting in a threshold value of 136.1 nA. Considering this and the fact that the highest measurement value was 150 nA, the determination threshold for the accuracy of the test (cut‐off value) was set to 150 nA. Measurements greater than or equal to 151 nA were classified as positive, while those less than or equal to 150 nA were classified as negative. This threshold ensured the NW‐Biosen's sensitivity and reliability in distinguishing between positive and negative results. To evaluate the high‐dose hook effect, a positive standard sample was prepared at a concentration 100 times the minimum detection limit in the commercial extraction buffer and then diluted 10‐fold to produce two concentrations. Each sample was repeatedly measured 20 times to assess consistency and accuracy (Figure 2C‐v).

### 2.3 Detection of SARS‐CoV‐2 Variants and Cross‐Reactivity Assessment With Recombinant Proteins, Respiratory Pathogens, Bacterial Strains, and Media Matrices Interferences

**FIGURE 3:**
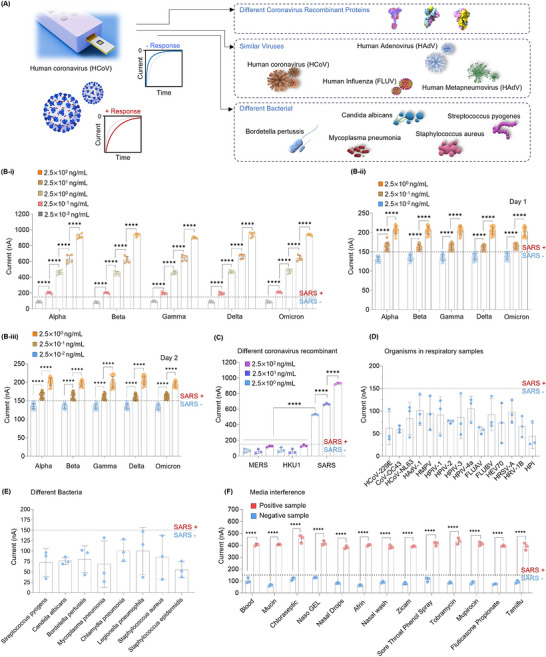
Cross‐reactivity with high‐priority pathogens from the same genetic family. (A) Schematic representation of a highly sensitive NW‐Biosen for pathogen detection. (B‐i) Dose‐dependent electrochemical responses to five SARS‐CoV‐2 variants (Alpha, Beta, Gamma, Delta, Omicron) (n = 5). (B‐ii, iii) Stability of NW‐Biosen responses on Day 1 and Day 2 in three concentrations, showing consistent detection for all variants (n = 20). (C) Signal response to recombinant proteins from other coronaviruses within the same genetic lineage (MERS, HKU1, SARS) at different concentrations (n = 3). (D) Assessment of cross‐reactivity with various organisms found in respiratory samples (n = 3). (E) Assessment of cross‐reactivity with potential bacterial contamination in eight different strains (n = 3). (F) Evaluation of potential media interference using 13 common substances and biological fluids (n = 3). All measurements were performed in TMB electrolyte using a mini‐potentiostat. Spiked samples were prepared by adding known concentrations of virus into negative patient swab samples that were confirmed by RT‐PCR. (Data = mean ± SD; **** *p* < 0.0001. One‐way ANOVA with Tukey for ≥3 groups; unpaired two‐tailed *t*‐test for 2 groups. Adjacent comparisons only; ns results not shown).

To experimentally verify the specificity of the NW‐Biosen, we tested a comprehensive panel that included five different SARS‐CoV‐2 variants, 15 clinically relevant respiratory viruses, eight bacterial species, and two recombinant coronavirus proteins: totaling 26 distinct pathogens and antigens. As illustrated in Figure 3A, this evaluation framework was designed to assess the biosensor's robustness within multiple potential sources of diagnostic interference. To further assess the performance of the NW‐Biosen, we evaluated its ability to detect the five major SARS‐CoV‐2 Variants of Concern (VOCs), including Alpha, Beta, Gamma, Delta, and Omicron, as designated by the World Health Organization [26]. Recombinant antigens representing each variant were spiked into RT‐PCR, which confirmed negative patient samples (Table S3), and were tested at multiple concentrations to assess dose‐dependent responses (Figure 3B‐i). For all five variants, the sensor showed a clear, monotonic, dose‐dependent increase in current over a 10^4^‐fold concentration range (2.5 × 10^−^^2^–2.5 × 10^2^ ng mL^−^^1^), with median signals rising from ∼100–300 nA at the lowest dose to ∼900–1000 nA at the highest; responses at ≥2.5 × 10^−^^1^ ng mL^−^^1^ were consistently above the 150 nA positive threshold and separated from SARS‐negative controls, with comparable magnitudes within variants and no systematic loss for Omicron. Additionally, to assess day‐to‐day stability, we re‐tested aliquots of the same variant‐spiked sample panel on separate days (Figure 3B‐ii, iii), and the currents were consistent between days with no systematic shift. As shown in Figure 3C, no detectable cross‐reactivity was observed with MERS‐CoV or HCoV‐HKU1 recombinant proteins. Currents remained at baseline and below the 150 nA cut‐off within the tested concentrations. In contrast, the SARS‐CoV‐1 recombinant protein produced a clear, dose‐dependent increase in current, exceeding the cut‐off at higher doses. We further tested a diverse group of clinically relevant respiratory viruses and bacterial species, including human coronaviruses (HCoV‐229E, HCoV‐OC43, HCoV‐NL63), influenza A and B viruses, respiratory syncytial virus (HRSV‐A), human metapneumovirus (HMPV), adenovirus (HAdV‐1), enterovirus 70 (HEV70), parainfluenza viruses (HPIV‐1 to HPIV‐4a), rhinovirus (HRV‐1B), and *Haemophilus parainfluenzae* (HPI). Additionally, we evaluated a broad panel of bacterial species, including *Streptococcus pyogenes*, *Staphylococcus aureus*, *Staphylococcus epidermidis*, *Candida albicans*, *Legionella pneumophila*, *Bordetella pertussis*, *Mycoplasma pneumoniae*, and *Chlamydia pneumoniae*, at clinically relevant concentrations (typically 1.0 × 10^6^ CFU/mL). As demonstrated in Figure 3D,E, none of these organisms produced a measurable electrochemical signal, indicating no significant cross‐reactivity. Furthermore, in silico analysis confirmed no detectable similarity between the SARS‐CoV‐2 nucleocapsid protein and proteins from *Mycobacterium tuberculosis* (n = 4151 sequences) or *Pneumocystis jirovecii* (n = 4993 sequences), supporting the specificity of the findings. A complete list of all tested organisms and their concentrations is available in Table S4. Samples for these experiments were produced by spiking the corresponding solution into the RT‐PCR negative patient samples (Table S5). To assess analytical specificity in different substances and biological fluids, we performed an interference study with blood, mucin, Chloraseptic, Naso GEL, saline nasal drops, Afrin, nasal wash, Zicam, sore‐throat phenol spray, tobramycin, mupirocin, fluticasone propionate, and Tamiflu (Figure 3F). RT‐PCR–confirmed negative patient matrices (Table S6) were tested with and without each interferent at the concentrations listed in Table S7. In each case, negatives remained below the 150 nA cut‐off (∼20–100 nA) and matched positives were well above it (∼350–500 nA), with no overlap within the decision line and no misclassifications; the separation was statistically significant.

### 2.4 Clinical Trials of NW‐Biosen for SARS‐CoV‐2 Antigen Detection in Clinical Samples

**FIGURE 4:**
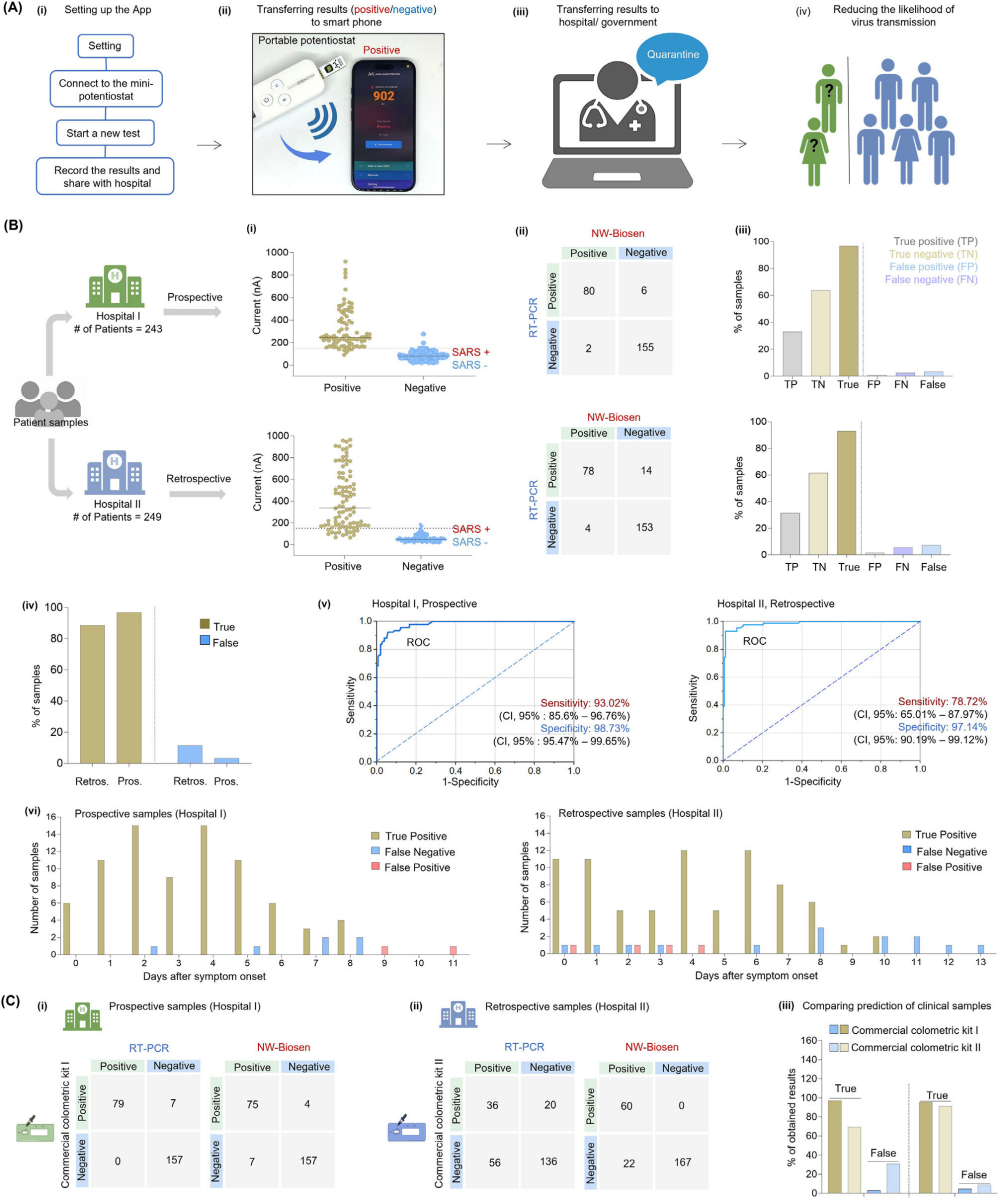
Clinical Trials of NW‐Biosen and Their Benchmarks. (A) Smartphone‐integrated biosensing platform for real‐time SARS‐CoV‐2 detection and infection control. (A‐i) Setting up the app involves connecting the mini‐potentiostat to a smartphone, initiating a new test, and recording results. (A‐ii) A photo of the portable mini‐potentiostat transmitting test results (positive/negative) to the smartphone for immediate feedback. (A‐iii) Test results are transferred to hospitals or government authorities. (A‐iv) Reducing the likelihood of virus transmission by ensuring timely isolation of infected individuals while awaiting confirmatory testing if necessary. Clinical samples were analyzed at two independent hospital sites using RT‐PCR as the reference standard. (B‐i) Scatter plot showing the distribution of current signals for RT‐PCR positive and negative samples. (B‐ii) Confusion matrices summarizing the diagnostic performance of the NW‐Biosen compared to RT‐PCR, with data from both retrospective and prospective samples. (B‐iii) Bar graphs depicting the percentage of true positives (TP), true negatives (TN), false positives (FP), and false negatives (FN) within both hospitals, based on a total of n = 492 samples. (B‐iv) Comparison of the overall rates of correct and incorrect classifications between retrospective and prospective sample groups. (B‐v) ROC curves illustrating the diagnostic accuracy of the sensor for the RdRp gene in hospital I (prospective) and hospital II (retrospective) sample groups. (B‐vi) Detection accuracy over time post symptom onset: Distribution of true positive, false negative, and false positive cases detected by NW‐Biosen relative to days post symptom onset in both Hospital I and Hospital II. (C) Clinical performance comparison of NW‐Biosen and commercial kits: (C‐i) confusion matrices showing the performance of RT‐PCR (actual values) and NW‐Biosen (predicted values) using prospective samples from Hospital I, compared to a commercial antigen kit I. (C‐ii) Same comparison for retrospective samples from Hospital II, evaluated against commercial kit II. (C‐iii) Percentage of true and false results obtained by commercial kits I and II when RT‐PCR and NW‐Biosen are used as references.

To enable rapid and reliable SARS‐CoV‐2 detection at the POC, we integrated the NW‐Biosen with a portable mini‐potentiostat containing a wireless data transfer system. Figure 4A‐i illustrates the testing process, which begins with a user‐friendly mobile application allowing users to configure the device, connect to the mini‐potentiostat, initiate a measurement, and record results. Once the electrochemical signal is acquired, the result, whether positive or negative, is automatically transferred in real time to the smartphone (Figure 4A‐ii). The results can then be shared directly with hospitals or health authorities (Figure 4A‐iii), and in case of a positive result, immediate action such as isolation measures can be initiated (Figure 4A‐iv). The portable and app‐based configuration supports decentralized and scalable testing, with smartphone integration enabling rapid data reporting. We further assessed the long‐term stability of the nanowell electrodes and the measurement consistency of the nanowell electrodes. The nanowell electrodes used 2 months later showed the same positive/negative discrimination as the electrode used immediately after manufacture (Figure S4). We evaluated measurement consistency between the benchtop CHI potentiostat and the mini‐potentiostat used for the POC testing. As shown in Figure S5A, positive and negative samples yielded comparable current responses on both instruments, indicating good instrument equivalence. Device‐to‐device variability in three mini‐potentiostats was also low, as evidenced by the bare nanowell electrodes showing similar current values (Figure S5B). Together, these results support the long‐term stability of the NW‐Biosen platform and the reliability of the miniaturized readout system for decentralized testing.

Following system integration, clinical validation was conducted using 492 patient samples from two independent hospitals. We ran study to confirm POC application under real workflows; the assay produced ∼10‐minute, sample‐to‐answer results with high levels of agreement with RT‐PCR. Figure 4B‐i shows a scatter plot of electrochemical current signals versus RT‐PCR results (targeting the RdRp gene). A total of 243 prospective samples from hospital I and 249 retrospective samples from hospital II were analyzed. At hospital I, the biosensor correctly identified 80 true positives and 155 true negatives, resulting in 96.7% accuracy with 8 misclassifications (2 false positives, 6 false negatives), as shown in Figure 4B‐ii, iii. At hospital II, 78 true positives and 153 true negatives were identified, corresponding to an accuracy of 92.8% with 18 misclassifications (4 false positives, 14 false negatives). Positive predictive value (PPV) and negative predictive value (NPV) were calculated from the confusion matrices to provide clinical measurements of test performance beyond sensitivity and specificity. In the prospective cohort, PPV and NPV were 97.56% and 96.27%, respectively, reflecting a high likelihood that both positive and negative results accurately represented true infection status. In the retrospective cohort, the PPV was 95.12%, and the NPV was 91.62%, with the slightly lower NPV being consistent with sample degradation associated with freeze–thaw handling. Figure 4B‐iv presents the summary of classification results that showed high accuracy with prospective samples. This is likely because prospective specimens were tested fresh, whereas retrospective samples had been frozen and later thawed before testing, which can compromise biomarker integrity. In addition, prospective cohorts benefit from standardized collection and handling procedure as well as more consistent clinical documentation, all of which could contribute to improved classification performance.

To evaluate diagnostic performance beyond a single threshold, ROC analysis was conducted using electrochemical current values ranging from ∼16 to 956 nA. An optimal threshold of 150 nA was selected as the fixed operational point of deployment. As shown in Figure 4B‐v, the biosensor achieved a sensitivity of 93.02% (95% CI: 85.6%–96.76%) and specificity of 98.73% (95% CI: 95.47%–99.65%) in the prospective group. In the retrospective group, sensitivity and specificity were 78.72% (95% CI: 65.01%–87.97%) and 97.14% (95% CI: 90.19%–99.12%), respectively. Using the fixed 150 nA cut‐off, sensitivity was higher in the prospective cohort, while specificity remained high in both cohorts (∼97–99%). The 95% CIs are narrow, indicating stable estimates in our n = 492 dataset. This data document high agreement with RT‐PCR using a single operating threshold. Figure 4B‐vi shows the distribution of test results over time post symptom onset, which are used to assess performance variation within time points. The majority of true positive results were observed within the first week after symptom onset, particularly between days 1–5, and are consistent with reports that antigen test sensitivity is highest early in infection when viral load peaks [27]. Figure S6A shows the relationship between RT‐PCR C_t_ values and days since symptom onset in positive clinical samples from both prospective and retrospective cohorts, and then shows false‐negative results identified by NW‐Biosen in both cohorts. C_t_ values exhibited a gradual increase with increasing days post symptom onset, consistent with a decline in viral load over time. However, false‐negative results identified by NW‐Biosen did not show a clear correlation with symptom onset day, indicating that the timing of testing alone did not bias the occurrence of false‐negative results. False positives were rare within all time points, confirming the high specificity of the assay, consistent with prior evaluations of SARS‐CoV‐2 antigen tests [28]. The relationship between RT‐PCR C_t_ values of clinical samples and current signals obtained from the NW‐Biosen for both prospective and retrospective cohorts is shown in Figure S6B. The false‐negative results detected by the biosensor were first observed at C_t_ = 22.7 and were predominantly observed at higher C_t_ values (C_t_ > 28), consistent with reduced viral load (there is an inverse relationship between C_t_ and viral load). In contrast, samples with lower C_t_ values showed higher current signals, indicating reliable sensor performance within the clinically relevant low‐to‐moderate C_t_ range. It was observed that among retrospective patient samples with C_t_ > 22.7, 37.8% showed false responses, whereas among prospective samples this was 15.8%, which is expected given the lower false‐response rate in prospective samples.

For benchmarking, the same clinical samples were tested using two commercially available COVID‐19 antigen kits. In the prospective group, the first commercial detection kit identified 75 true positives and 157 true negatives, totaling 232 correct results (95.5% accuracy) and 11 incorrect results (4 false positives, 7 false negatives) (Figure 4C‐i). In the retrospective group, NW‐Biosen achieved 78 true positives and 153 true negatives, with 231 correct results (92.8% accuracy) and 18 incorrect results (4 false positives, 14 false negatives) (Figure 4C‐ii). In comparison, the second commercial detection kit yielded 60 true positives and 167 true negatives, totaling 227 correct results (91.2% accuracy), with 22 false negatives and no false positives. These benchmarking results are summarized in Figure 4C‐iii. On identical samples, NW‐Biosen showed higher overall accuracy than both commercial antigen kits with fewer total errors.

In our previous studies, nanowell electrodes have been successfully used to detect nucleic‐acid targets and diverse protein biomarkers, including nucleic acids (DNA oligonucleotides) and protein biomarkers such as carcinoembryonic antigen (CEA), alpha‐fetoprotein (AFP), and alkaline phosphatase (ALP) [8, 9, 29, 30]. To further support platform‐level adaptability for monitoring other biomarkers, we performed biosensing experiments demonstrating the detection of an additional clinically relevant biomarker, cardiac troponin I (TnI), using semiconductor‐fabricated nanowell electrodes functionalized with DTSSP surface chemistry. The detection was performed label‐free using TMB or a ferrocyanide/ferricyanide mixture as the redox electrolyte, with a dynamic range of 1–1000 pg/mL for TnI, using EIS or SWV techniques (Figure S7). Therefore, the NW‐Biosen platform can be readily adapted to non‐viral protein biomarkers by simply changing the capture antibody, without altering the nanowell geometry or fabrication workflow, demonstrating its generalizability to other infectious or non‐infectious disease biomarkers.

## 3 Discussion

Multiple diagnostic modalities have been developed to detect SARS‐CoV‐2, including electrochemical sensors, optical and colorimetric assays, magnetic chemiluminescent immunoassays, liquid chromatography‐tandem mass spectrometry, isothermal nucleic‐acid tests (reverse‐transcription loop‐mediated isothermal amplification and CRISPR–Cas), and selected label‐free photonic devices. Table S8 summarizes clinically evaluated non‐electrochemical systems, and Tables S9 and S10 assemble the electrochemical platforms. In practice, assays are sorted by test setting (central laboratory versus point of care) and analyte class (viral RNA, viral antigen, or host antibody). Central‐laboratory methods such as magnetic chemiluminescent immunoassay and liquid chromatography‐tandem mass spectrometry provide high accuracy and throughput but require complex instruments and controlled workflows. POC optical lateral‐flow tests and electrochemical sensors minimize the time required to gather results and infrastructure needs, but performance can vary with sample type and lot‐to‐lot differences. Practical accuracy is driven by sample type, target selection, and standardized procedures (calibration and cross‐reactivity testing), not only by analytical detection limits. Electrochemical platforms frequently use nano‐ and micro‐structured materials to strengthen analytical performance. Larger effective surface areas and improved receptor orientation can increase the capture efficiency and signal, enabling rapid and quantitative readouts. However, electrochemical platforms are particularly vulnerable to non‐specific biomolecular adsorption and biofouling in complex biological matrices (e.g., blood, saliva), often resulting in false positives, signal drift, or poor reproducibility [31]. Additionally, labor‐intensive fabrication processes pose difficulties in reproducible fabrication, including inconsistent nanostructure geometry and batch‐to‐batch uniformity, as well as high manufacturing costs and scalability challenges, which challenge affect quality control and obstruct clinical translation [32]. Tables S9 and S10 examine these electrochemical implementations in detail, highlighting the specific nanomaterial choices and mitigation strategies, and situates this work (nanowell electrochemical assay with CA readout) as a practical path to POC use through wafer‐scale manufacturing and prospective clinical validation. Table S9 compares NW‐Biosen with other electrochemical COVID‐19 biosensors and shows that most prior systems lack large‐scale clinical validation, standardization, or reproducible fabrication. Table S10 extends this comparison to nanostructured electrochemical biosensors and shows that, whereas most rely on randomly grown architectures such as nanoflowers, nanotubes, or dendritic films, these approaches face inherent limitations in reproducibility and manufacturability due to uncontrolled geometry.

We developed NW‐Biosen as a nanowell‐based biosensor that confines the active sensing area to precisely engineered nanowells. Insulating, non‐adsorptive materials surround these nanostructured wells, minimizing non‐specific interactions and improving signal‐to‐noise ratios in complex samples. This design enhances biomolecule orientation and reduces steric hindrance, thereby preventing biofouling. Unlike many conventional nanomaterial‐based systems, NW‐Biosen leverages semiconductor‐compatible processes for wafer‐scale fabrication, delivering both high sensitivity and manufacturing scalability. We employed a high‐throughput semiconductor manufacturing workflow, capable of fabricating over 1000 nanowell sensors per 8‐inch wafer with excellent uniformity. Nanowell arrays with various diameters (from 230 to 500 nm) were patterned using a two‐step process involving thin‐film metal deposition and reactive ion etching. Our fabrication strategy utilized a KrF scanner lithography system, enabling sub‐micron resolution within large wafer areas with high reproducibility. In contrast to slower and lower‐throughput methods like electron‐beam lithography or focused ion beam milling, our process supports scalable production without compromising precision or electrochemical performance, which can maintain high‐quality electrodes while reducing manufacturing costs [33]. This consistency is crucial for achieving regulatory approval and actual diagnostic reliability.

To support POC deployment, the biosensor was integrated with a portable mini‐potentiostat and smartphone application for real‐time data acquisition and transmission. This system enables decentralized testing, streamlined reporting, and rapid outbreak response through immediate result sharing with healthcare providers and public health agencies. Its modular design allows for easy reconfiguration for other targets by replacing the biorecognition element (Figure S7). Clinical validation was performed using 492 samples from two hospitals, yielding 96.7% accuracy in prospective samples and 92.8% in retrospective samples. The number of clinical samples tested places this study among the largest patient‐evaluated nanostructure‐based biosensor reports to date. Testing different variants of the virus, as well as lot‐to‐lot and day‐to‐day testing, showed consistent results from the sensors, which confirms reproducibility. The ROC analysis confirmed an optimal threshold of 150 nA. Cohen's kappa values of 0.927 and 0.841 in the respective cohorts indicated near‐perfect agreement with RT‐PCR, outperforming standard commercial antigen kits. These results emphasize NW‐Biosen's readiness for patient sample diagnostics. In contrast to many biosensors that lack scalability or validation beyond lab conditions, NW‐Biosen combines wafer‐scale manufacturing, high sensitivity, and clinical‐grade specificity. Its performance was validated in both prospective and retrospective samples from independent hospitals, with rapid 10‐minute sample‐to‐answer results using minimally invasive nasal swabs. The assay is fast, and although it is not the fastest, its portable ∼10‐minute workflow places it among leading POC options, and it compares favorably with typical molecular and analyzer‐based methods that require longer total processing times. Meanwhile, compared to commercially available kits, it offered lower false‐negative and false‐positive rates. With its scalable, modular architecture, NW‐Biosen provides a platform for next‐generation diagnostics. Its adaptability to new targets, strong clinical performance, and digital integration make it well‐suited for emerging infectious diseases, chronic conditions, and personalized medicine. This study highlights the potential of semiconductor‐inspired nanofabrication to bridge the gap between biosensor development and clinical deployment.

To further improve the clinical accuracy of the NW‐Biosen, future studies should combine advanced signal analytics with stronger manufacturing controls. On the analytical side, machine‐learning models can use features from chronoamperograms to suppress noise, correct baseline drift, and adapt decision thresholds to different sample types. Training should use multi‐site data sets with calibration‐transfer and external validation, so the model remains reliable in new clinics. On the hardware and processing side, enhanced semiconductor manufacturing (stepper‐based lithography with tighter critical‐dimension control, uniform gold surface morphology, and inline metrology) can reduce well‐to‐well and lot‐to‐lot variation even more. Standardized surface chemistry (oriented receptor immobilization, low‐fouling linkers, and robust passivation) together with built‐in controls (duplicate wells, reference electrodes, and ratiometric readouts) can also stabilize signals. In the future, comprehensive, long‐term stability and storage‐dependence performance, which are essential factors for the clinical application of the POC system and its commercialization, need to be further evaluated.

## 4 Conclusion

Here, we developed NW‐Biosen, a semiconductor‐manufactured nanowell biosensor designed to enable scalable wafer‐level fabrication and support practical clinical implementation. The electrode array was fabricated through a wafer‐scale lithography process that produced uniform nanowell structures with high batch‐to‐batch consistency, enabling mass production of more than 1000 sensors per wafer. Electrochemical characterization confirmed stable ohmic behavior for the bare sensors and reproducible CV, SWV, and EIS responses throughout the functionalization process, demonstrating reliable and consistent sensor performance. The biosensor showed sensitive SARS‐CoV‐2 antigen detection within minutes using a CA‐based readout, with consistent performance between lots and over repeated testing. Biosensors maintained analytical selectivity in the presence of SARS‐CoV‐2 variants, related coronavirus proteins, common respiratory pathogens, bacterial species, and potential interfering substances, supporting their robustness in diverse sample conditions. Clinical validation with 492 patient samples, including prospective and retrospective cohorts, showed high agreement with RT‐PCR results and fewer misclassifications than commercial antigen kits, supporting NW‐Biosens's suitability for the POC testing. Consistent diagnostic performance with patient samples that were collected and tested on different days further supports the practical robustness of the sensing platform against day‐to‐day variability in sample quality, including potential matrix‐induced fouling under routine clinical operation. Integration with a portable potentiostat and wireless data transfer to smartphones enabled rapid result reporting and real‐time communication with healthcare personnel. Beyond SARS‐CoV‐2, the nanowell architecture and semiconductor‐based manufacturing approach provide a pathway for developing scalable, adaptable biosensors for infectious or non‐infectious diseases and other clinically relevant biomarkers.

## 5 Experimental Section

### 5.1 Reagents

The inactivated SARS‐CoV‐2 virus (BetaCoV/Korea/KCDC03/2020, NCCP43326), mouse‐derived monoclonal antibody targeting the SARS‐CoV‐2 virus (40591‐MM43), and the horse radish peroxidase (HRP) conjugated antibody from mouse derived monoclonal antibody (40150‐D001‐H) were purchased from Sino Biological US Inc. (Wayne, PA, USA). Details of the suppliers for other viruses and bacteria are provided in Table S3. The extraction buffer was obtained from Miri Medix company (Daejeon, South Korea). Carbonate‐bicarbonate buffer, human cardiac TnI enzyme‐linked immunosorbent assay (ELISA) kit, TMB, and DTSSP were purchased from Thermo Fisher Scientific (Waltham, MA, USA). Potassium ferricyanide (III), potassium ferrocyanide (II), ethanolamine hydrochloride, PBS, Tween 20, Triton X‐100, BSA, and KCl were obtained from Sigma‐Aldrich (St. Louis, MO, USA).

### 5.2 Fabrication and Characterization of Nanowell Electrodes

The nanowell sensor was fabricated using conventional semiconductor photolithography techniques. An oxidized 8‐inch silicon wafer served as the substrate for sequential deposition of thin films: 20 nm of Ti, 200 nm of Au, and 200 nm of SiO_2_. These layers were deposited using an evaporator and a plasma‐enhanced chemical vapor deposition (PECVD) system to ensure uniform coverage and adhesion. A photoresist (PR) layer was spin‐coated onto the wafer, followed by ultraviolet (UV) exposure through a photomask including the top view of nanowell patterns. PR development and subsequent dry etching were employed to etch the SiO_2_ layer and create the nanowell structures, selectively exposing the Au surface for immobilization processes. The nanostructures of the electrodes were analyzed using a scanning electron microscope (SEM, Hitachi S‐4800). The sensing area was integrated into a USB‐compatible printed circuit board (PCB) substrate with copper clad laminate. The mini‐potentiostat (Esens2000) with wireless data transfer function was obtained from MARA Nano Tech company (Daejeon, South Korea), which operates in CA mode and has been specifically customized to accommodate a USB‐type sensor.

### 5.3 Functionalization of Nanowell Electrodes

The nanowell electrode was incubated with 40 µL of 5 mm DTSSP in DI water for 2 h at room temperature. After washing with PBS and drying, 100 µg/mL of antibody in PBS was applied and incubated at 37°C for 1 h. Unreacted sites were blocked using a blocking buffer (1% BSA and 0.1% Triton X‐100 in PBS) for 30 min, followed by a final washing and drying step. For detection of cardiac TnI, the electrodes were functionalized with DTSSP. The anti‐TnI antibody was obtained from a commercial ELISA kit prepared according to the manufacturer's instructions and then incubated on the electrode surface for 2 h at room temperature. Ethanolamine (10 mm in PBS, 30 min) was used as the blocking reagent. TnI dissolved in PBS was incubated with the electrodes for 30 min.

### 5.4 Electrochemical Measurement

The electrochemical performance was evaluated using a CHI 660E workstation (CH Instruments, Bee Cave, TX, USA) to detect CV, SWV, and EIS in a redox solution of 5 mm potassium ferricyanide (III) dissolved in 0.1 m KCl, and 0.1 m PBS (pH 7.2). CV was conducted with a potential range of −0.6 to 0.6 V, a scan rate of 0.05 V/s, and a sample interval of 0.001 V. SWV used the same potential range, with a step increment of 0.005 V, amplitude of 0.025 V, and frequency of 10 Hz. EIS measurements were performed from 16 to 0.01 Hz at an amplitude of 0.01 V. For clinical sample detection, the portable mini‐potentiostat was used for CA measurements in TMB as the redox substrate. A 5‐minute TMB incubation consistently produced strong and reliable redox signals. The assay was run at an initial potential of 0 V and a low potential of −0.05 V, with a 10‐second pulse width, 0.05‐second sample interval, 2‐second quiet time, and 1e‐6 A/V sensitivity.

To assess device‐to‐device variability of the mini‐potentiostat, three individual mini‐potentiostats were used to measure the bare signal of three nanowell electrodes using TMB as the redox electrolyte. Electrochemical measurements using EIS or SWV for the detection of TnI were performed with a CHI instrument using TMB or 5 mm ferrocyanide/ferricyanide in PBS as label‐free detection reagents.

### 5.5 In Vitro Sample Preparation

The sample used for in vitro testing consisted of specific antigens from the SARS‐CoV‐2 nucleocapsid protein. To test and optimize electrode development in the lab, the inactivated SARS‐CoV‐2 virus was used. The virus was inactivated with 0.5% Triton X‐100 and was diluted with a negative standard (0.05% Tween 20 in PBS (PBST)) substance to prepare samples at various concentrations. These samples were analyzed using the Allplex SARS‐CoV‐2 Assay (Seegene, South Korea), an RT‐PCR method, to confirm their cycle threshold (C_t_) values. For positive tests, a specific concentration of antigen was added to the mixture, while PBST was used as the standard for negative controls. The HRP‐conjugated detection antibody was diluted to 1 µg/mL in PBST and mixed at a 1:1 (v/v) ratio with 1% sodium carbonate solution. The sample was then combined with a secondary antibody (HRP‐conjugated antibody) at a 1:1 (v/v) ratio. A volume of 25 µL of this prepared solution was applied to the electrode, and the reaction was allowed to proceed for 5 min at room temperature. Afterward, the electrode surface underwent a washing step, which involved either gently wiping it with a cotton swab or more rigorously washing it with PBST. The surface was then dried using a blower. For detection, 25 µL of TMB was applied to the electrode and allowed to react for 5 min at room temperature. The electrochemical measurements were performed using the mini‐potentiostat. The mini‐potentiostat was configured to display reduction (negative) currents as positive values for convenience. User‐friendly instructions for using the mini‐potentiostat are provided in the Supporting Information.

### 5.6 Clinical Specimen Collection

Nasopharyngeal or anterior nasal samples were collected in accordance with the instruction manual, adhering to the Centers for Disease Control and Prevention (CDC) interim guidelines for collecting, handling, and testing clinical specimens from people for COVID‐19 [34]. Sterile swabs were used to ensure proper sample collection, and samples were processed immediately to maintain integrity and prevent degradation. Clinical performance tests were conducted at a single institution in a single‐blind manner to evaluate the performance of the nanowell biosensing kit. Adult men and women over the age of 19, regardless of COVID‐19 symptoms, who provided written consent were included. Positive and negative study subjects were selected based on RT‐PCR results. This rigorous selection process ensured reliable sample quality for performance evaluation. The study included a combination of prospective samples (n = 243 from Korea Institute of Radiological & Medical Sciences, hospital I) and retrospective samples (n = 249 from Daejeon Eulji Medical Center, hospital II). In total, the study evaluated 492 samples, comprising 178 SARS‐CoV‐2‐positive patients and 314 SARS‐CoV‐2‐negative individuals. Retrospective samples were collected from previously confirmed COVID‐19 cases to validate the kit's accuracy using historical data, while prospective samples reflect ongoing testing, ensuring that the biosensor's performance is applicable to both past and current clinical scenarios. Furthermore, the performance of the proposed biosensing kit was compared with two commercially available diagnostic kits: Asan Easy Test COVID‐19 Ag and SGTi‐flex COVID‐19 Ag.

### 5.7 Validating Biosensor Results via RT‐PCR

The Allplex SARS‐CoV‐2 Assay (Seegene, South Korea) was performed following the manufacturer's instructions using RT‐PCR to detect SARS‐CoV‐2 RNA in clinical samples. The process began with RNA extraction from clinical samples, such as nasal or nasopharyngeal swabs, to isolate viral genetic material. The extracted RNA was then converted into complementary DNA (cDNA) using the enzyme reverse transcriptase. The cDNA was amplified using primers and fluorescent probes specific to SARS‐CoV‐2 genes, including the E (envelope), RdRp (RNA‐dependent RNA polymerase), and N (nucleocapsid) genes.

### 5.8 Cross‐Reactivity Analysis

To investigate the potential cross‐reactivity of the electrochemical nanowell biosensing kit, an in silico analysis was performed using the BLAST, provided by the National Center for Biotechnology Information (NCBI) [35]. BLAST is a bioinformatics tool that compares protein sequences to identify regions of similarity (homology) between a target protein and other protein databases. The SARS‐CoV‐2 nucleocapsid protein sequence (used as the target) was compared with the protein sequences of various microorganisms, including *Human coronavirus HKU1*, *Mycobacterium tuberculosis*, and *Pneumocystis jirovecii*. The results were analyzed to identify any significant homology that could indicate a risk of cross‐reactivity.

### 5.9 Data and Statistical Analysis

Statistical analyses were performed in GraphPad Prism 8 to analyze and interpret data generated by the biosensors. For comparisons involving two independent groups, unpaired two‐tailed *t*‐tests were used. For analyses with three or more groups, one‐way ANOVA followed by Tukey's multiple‐comparisons test was used. Data were reported as mean ± SD. Significance was denoted as ns (*p* ≥ 0.05), * (*p* < 0.05), ** (*p* < 0.01), *** (*p* < 0.001), and **** (*p* < 0.0001). To calculate the ROC curve, the biosensor's output values were ordered from lowest to highest. At each threshold, samples were classified as positive or negative, and corresponding sensitivity (TP / (TP + FN)) and false positive rate (FP / (FP + TN)) values were calculated. The threshold that provided the best trade‐off between sensitivity and specificity was selected. These pairs were plotted to generate the ROC curve [36]. Confidence intervals (CIs) for sensitivity and specificity were calculated using the Wald method for binomial proportions at a 95% confidence level. The formula used was:

(1) $$CI=p\pm z\sqrt{\frac{p\left(1-p\right)}{n}}$$

where, *p* is the observed proportion (e.g., sensitivity = TP / (TP + FN)), *n* is the total number of cases, and *z* is the z‐score corresponding to the desired confidence level (1.96 for 95% CI).

Positive predictive values (PPVs) and negative predictive values (NPVs) were calculated to assess the clinical interpretability of the biosensor results. PPVs were defined as TP / (TP + FP), and NPVs as TN / (TN + FN), where TP, FP, TN, and FN represent true positives, false positives, true negatives, and false negatives, respectively [37]. To account for the prevalence‐dependent nature of PPVs and NPVs, additional calculations were performed using Bayes’ theorem, incorporating sensitivity, specificity, and assumed disease prevalence. PPVs and NPVs were estimated across a range of clinically relevant prevalence values to reflect potential real‐world screening and diagnostic scenarios.

Cohen's kappa coefficients were calculated to assess diagnostic agreement between the biosensor and RT‐PCR. The coefficient is defined as:

(2) $$\kappa =\frac{\left(\mathrm{Po}-\mathrm{Pe}\right)}{\left(1-\mathrm{Pe}\right)}$$

where 𝑃_𝑜_ is the actual agreement between the two tests, and 𝑃_𝑒_ is the agreement expected by chance based on their individual result distributions.

## Author Contributions

**Yoo Min Park**: methodology, investigation, writing – original draft. **Zahra Rezaei**: investigation, writing – original draft. **Nam Ho Bae**: conceptualization, supervision, funding acquisition. **Donggee Rho**: methodology, investigation. **Da‐Seul Kim**: writing – original draft. **EunYoung Go**: methodology, investigation. **YoungTae Seo**: methodology. **Seok Jae Lee**: methodology, investigation, project administration. **Won Chan Seo**: methodology. **Luke P. Lee**: project administration. **HeaYeon Lee**: conceptualization, supervision, funding acquisition, project administration, writing – review and editing. **Su Ryon Shin**: conceptualization, supervision, funding acquisition, project administration, writing – review and editing.

## Funding

This work was supported by the National NanoFab Center (NNFC) through the Nano Medical Device Project (Ministry of Science and ICT) (Y.M.P., N.H.B.); the Global Semiconductor Advanced Fab Utilization Project (Ministry of Science and ICT) (N.H.B.); the Technology Innovation Program (grants 20015577, 20025649, RS‐2022‐00154855, RS‐2024‐00508418, and RS‐2024‐00438660) funded by the Ministry of Trade, Industry and Energy (MOTIE, Korea) (Y.M.P., N.H.B.); the Government‐wide R&D Program for Infectious Disease Prevention and Control (grant RS‐2023‐KH140543) (Y.M.P., H.Y.L.); the Nano & Material Technology Development Program of the National Research Foundation of Korea (NRF), funded by the Ministry of Science and ICT (grant RS‐2024‐00444177) (D.R., S.L.); and the NNFC, funded by the Ministry of Science and ICT (grant RS‐2024‐00440903) (H.Y.L., S.R.S.). This research was also supported by the National Research Council of Science & Technology (NST) grant funded by the Ministry of Science and ICT (No. GTL25061‐000), and by the Development of a Strategic Platform to Support Bio–Semiconductor Convergence Technologies and Services Program of the NNFC.

## Ethics Statement

This study was approved by the Institutional Review Board under two protocols (IRB File No. 2022‐03‐004‐004 and IRB File No. 2022‐10‐011) from the Korea Institute of Radiological and Medical Sciences (Hospital I) and Daejeon Eulji Medical Center (Hospital II), respectively. Human specimens were obtained from two sources: prospectively collected samples from participants at the Korea Institute of Radiological and Medical Sciences who provided written informed consent prior to participation, and retrospective de‐identified clinical specimens from Daejeon Eulji Medical Center, for which the requirement for additional consent was waived. All procedures were performed in accordance with institutional regulations and the Declaration of Helsinki.

## Conflicts of Interest

The authors declare no conflicts of interest.

## Supporting information




**Supporting File**: advs74693‐sup‐0001‐SuppMat.docx.

## Data Availability

All data supporting the findings of this study are available from the corresponding authors upon request.
