# Supplementary material for: Mass‐Produced and High‐Performance Nanowell Biosensor Fabricated via Semiconductor Manufacturing for Rapid and Accurate COVID‐19 Diagnosis in the Clinical Field
Source: Adv Sci (Weinh). 2026 Mar 12;13(33):e22897. doi: 10.1002/advs.202522897 (PMC13271615; doi:10.1002/advs.202522897)
Supplement: Supplementary file 1 — Supporting File: advs74693‐sup‐0001‐SuppMat.docx. [file ADVS-13-e22897-s001.docx]

SUPPORTING INFORMATION

Mass-Produced and High-Performance Nanowell Biosensor Fabricated via Semiconductor Manufacturing for Rapid and Accurate COVID-19 Diagnosis in the Clinical Field

*Yoo Min Park^†^, Zahra Rezaei^2†^, Nam Ho Bae, Donggee Rho, Da-Seul Kim, EunYoung Go, YoungTae Seo, Seok Jae Lee, Won Chan Seo, Luke P. Lee, HeaYeon Lee^*^, and Su Ryon Shin^*^*

Y.M. Park, N.H. Bae, D. Rho, S. Lee

Center for Nano-Bio Development, National NanoFab Center (NNFC); 291 Daehak-ro, Yuseong-gu, Daejeon 34141, Republic of Korea

Z. Rezaei, D.S. Kim, L.P. Lee, H.Y. Lee, S.R. Shin

Division of Engineering in Medicine, Brigham and Women’s Hospital, Department of Medicine, Harvard Medical School; Cambridge, Massachusetts 02139, United States

E.Y. Go, H.Y. Lee

Mara Nanotech INC., Hanmir Hall, Yongdang Campus, Pukyong National University; Nam-gu 48548, Republic of Korea

Y.T. S, H.Y. Lee

Mara Nanotech New York INC.; 1 Pennsylvania Plaza, Suite 1423, New York, NY 10119, USA

W.C. Seo

Department of Materials System Engineering, Pukyong National University, Nam-gu 48513, Republic of Korea

^†^ The authors contributed equally to this work.

^*^ Co-correspondences: [h.lee@maranano.com](mailto:h.lee@maranano.com) (H.Y.L.), [sshin4@bwh.harvard.edu](mailto:sshin4@bwh.harvard.edu) (S.R.S.)


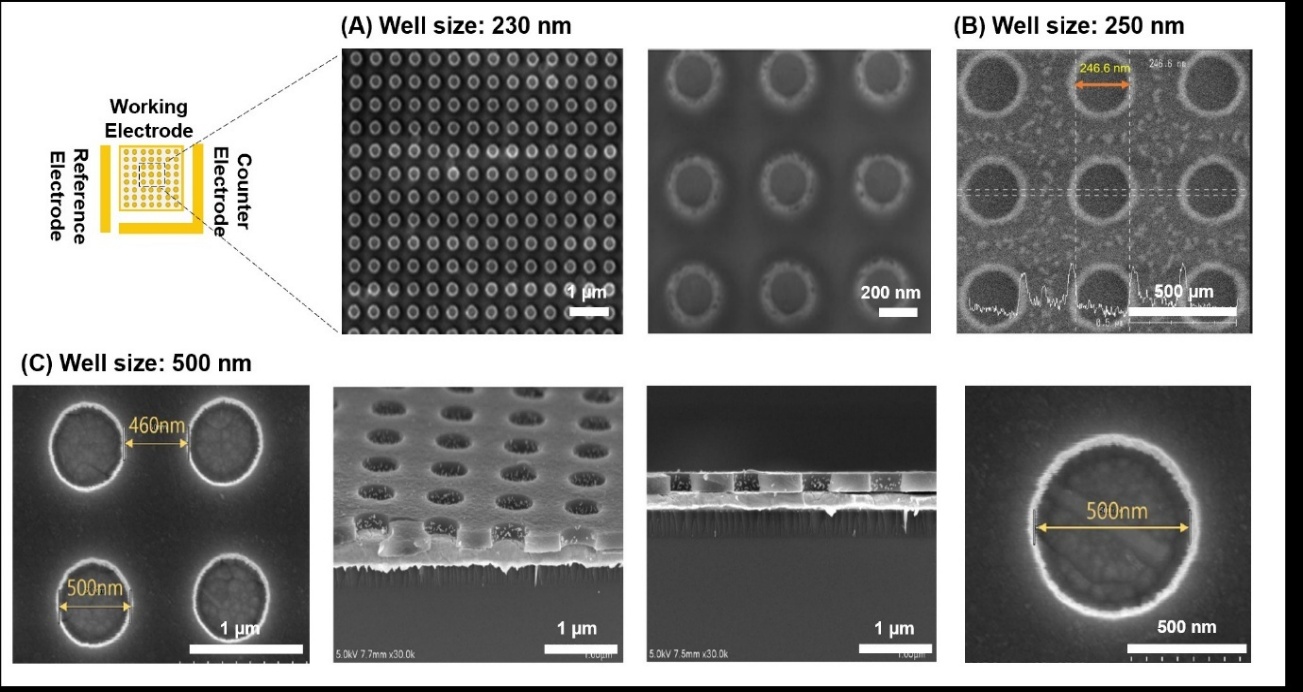


Figure S1. SEM images of nanowell array electrode surfaces with varying well sizes: (A) 230 nm, (B) 250 nm, and (C) 500 nm. The images illustrate well-defined, uniform nanowell geometries and surface topographies within different dimensions, confirming the precision and reproducibility of the fabrication process.


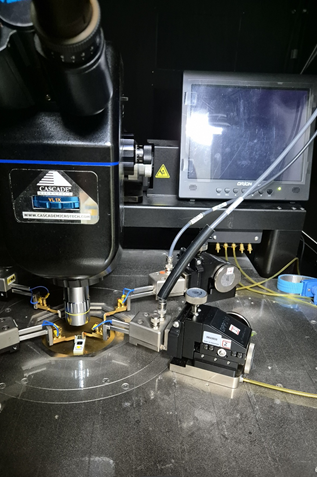


Figure S2. The prepared nanowell sensor’s resistance was measured using KEITHLEY 4200-SCS.

*
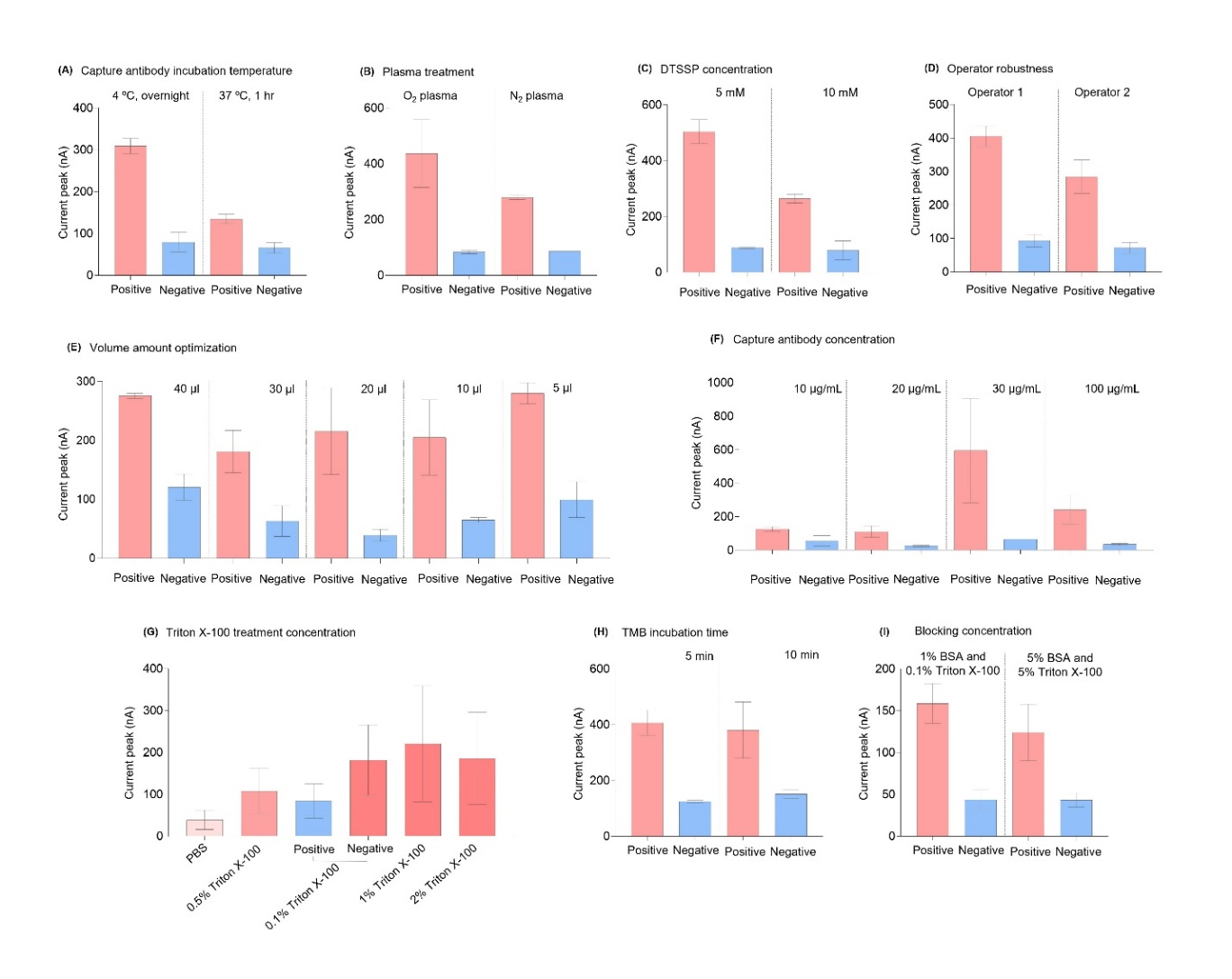
*

Figure S3. Optimization of parameters for biosensing: (A) Capture antibody incubation temperature (n = 2), (B) surface plasma treatment (O₂ vs N₂, n = 2), (C) DTSSP concentration (n = 2), (D) operator robustness (positive n = 3, negative n = 9), (E) sample loading volume (n = 2), (F) capture antibody concentration, (G) Triton X-100 concentration in virus treatment (PBS n = 2; 0.1% Triton X-100 n = 2; other Triton X-100 concentrations n = 4, except 0.5% Triton X-100 with n = 15 for both positive and negative), (H) TMB incubation time (n = 2), and (I) blocking buffer concentration (n = 3). Data=mean ± SD.


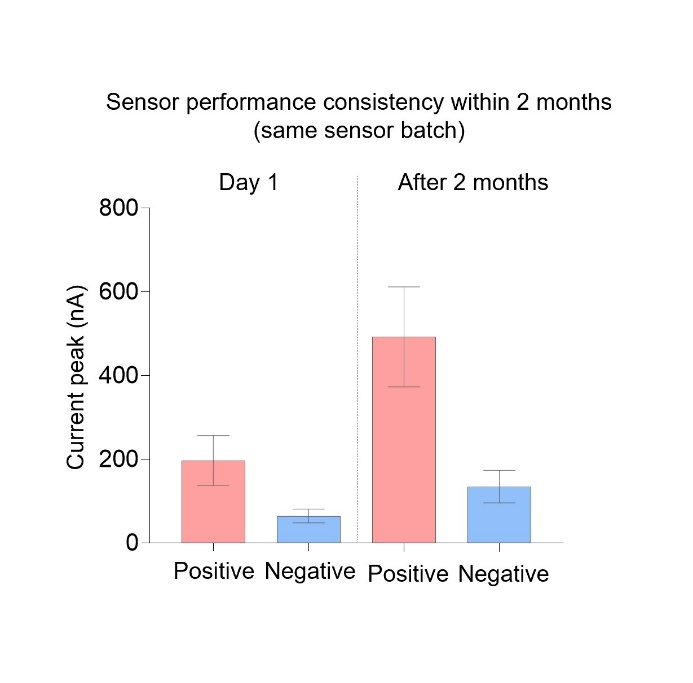


Figure S4. Performance consistency of sensors from the same wafer over 2 months. Data are shown as mean ± SD (n = 4).


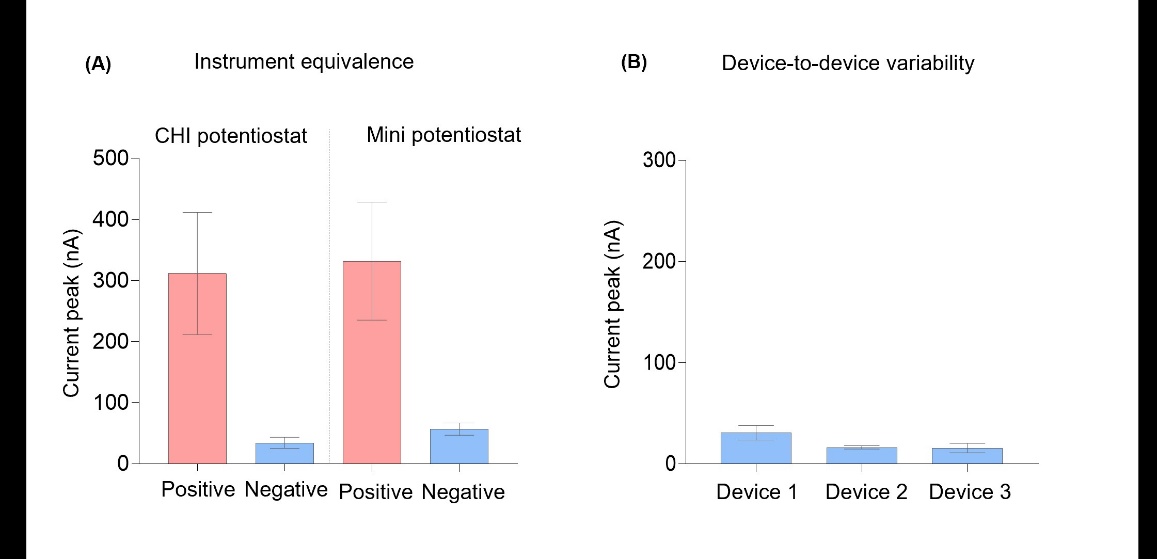


Figure S5. (A) Instrument equivalence between a benchtop CHI potentiostat and the mini-potentiostat. Signals for positive and negative samples were measured using five mini-potentiostat units (data = mean ± SD, n = 5). (B) Device-to-device variability in three mini-potentiostat. The current peak was obtained using bare nanowell electrodes in TMB electrolyte solution (data = mean ± SD, n = 3 (nanowell electrode)).


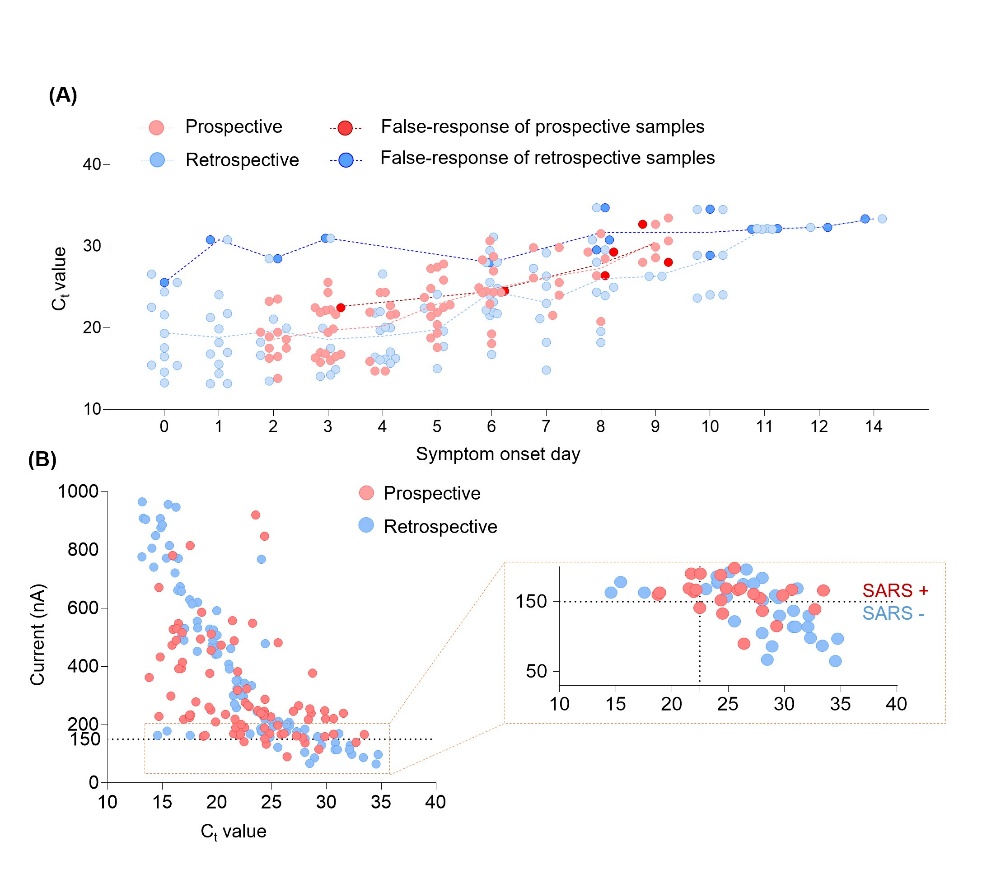


Figure S6. (A) Relationship between RT-PCR C_t_ values (RdRp gene) of positive clinical samples from Hospital I and Hospital II with days since symptom onset for prospective and retrospective groups. False-negative results of the NW-Biosen are highlighted for each group. Dotted lines indicate temporal trends in the average C_t_ values. (B) Correlation between RT-PCR C_t_ values and NW-Biosen platform current.


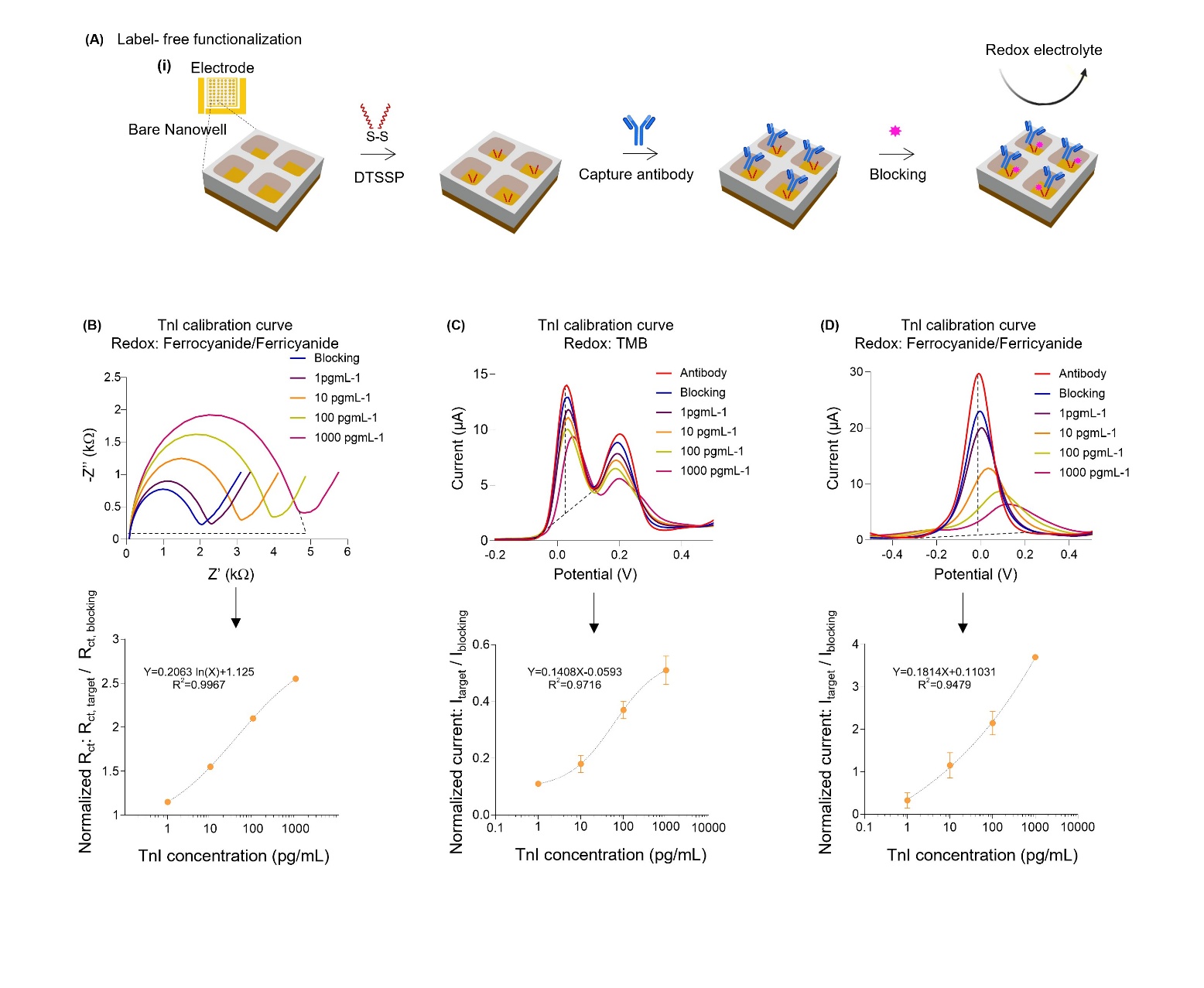


Figure S7. Label-free nanowell biosensing of cardiac TnI: (A) Schematic of surface functionalization and label-free detection on nanowell electrodes. (B–D) Electrochemical responses and calibration curves for TnI detection in PBS using EIS and SWV readouts with different redox systems (data = mean ± SD, n = 2).

Instruction of Using Electrochemical Nanowell Array Biosensing Kit

Sample extraction

All samples were brought to room temperature (15–25°C) before processing. For sample pre-treatment, patients were instructed to insert the cotton swab up to 1.5 cm into both nostrils and rotate it in a circular motion at least five times to ensure adequate sample collection. The collected swab was then thoroughly mixed with the extraction buffer to facilitate sample extraction. To maximize recovery, both sides of the tube were pressed to squeeze out the swab contents completely. The nozzle cap was securely closed, and the sample was allowed to rest at room temperature for 10 minutes before further processing (Figure S8).


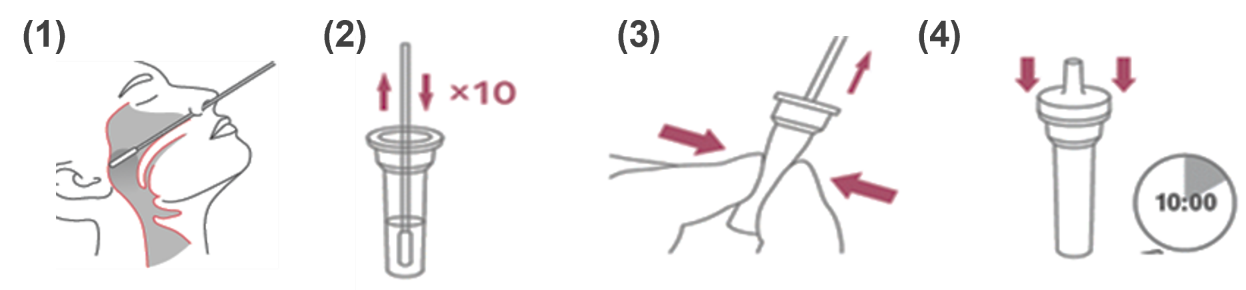


Figure S8. Illustration of the sample pre-treatment steps: (1) Collection of the nasal swab sample by inserting the swab up to 1.5 cm into both nostrils and rotating it in a circular motion at least five times. (2) Mixing the swab in the extraction buffer by moving it up and down at least ten times. (3) Pressing the sides of the extraction tube to squeeze the swab for thorough sample recovery. (4) Closing the nozzle cap and leaving the tube at room temperature (15–25°C) for 10 minutes to complete the extraction process.

Sample Application on Electrode Surface

One drop of the extracted sample was carefully deposited onto the electrode surface and allowed to incubate for 5 minutes. To remove the solution, the device was tilted at a 15-degree angle, and the solution was absorbed using a swab. The swab was then rolled over the device surface to ensure complete absorption of any residual solution. Finally, the black edges of the sample injection area were wiped clean with an unused cotton swab to remove any remaining traces of the solution. These steps are illustrated in Figure S9.


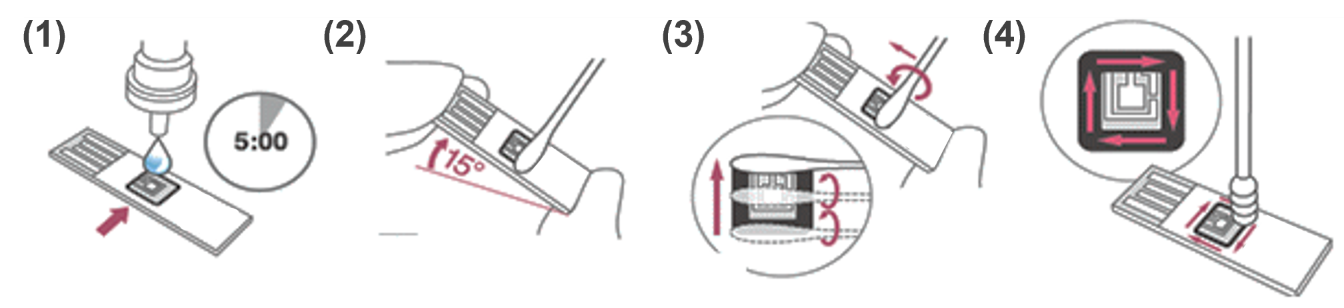


Figure S9. Illustration of the detection process: (1) Depositing one drop of the extracted sample onto the electrode surface and allowing it to incubate for 5 minutes. (2) Tilting the device at a 15-degree angle to facilitate the removal of the sample solution. (3) Rolling a swab gently over the electrode chamber to absorb any remaining solution without touching the electrode surface. (4) Wiping the edges of the sample injection area with an unused cotton swab to ensure all residue is removed and the surface is clean for subsequent analysis.

Measurement Using Mini-Potentiostat

The sensor was first inserted into the mini-potentiostat (measuring instrument). One drop of the extracted solution was deposited onto the sensor, which was then left at room temperature for 5 minutes. To initiate the measurement, the power button on the instrument was pressed and held for 3 seconds to turn it on. The measurement was started by pressing the measurement button, and after 10 seconds, the result was observed. The process is illustrated in Figure S10. The result was measured 5 minutes after dropping the detection solution and was considered valid; measurements taken beyond this time were deemed unreliable. If "P" appeared on the screen, the sample was deemed positive for the COVID-19 virus antigen, indicating a measurement greater than or equal to 151. If "n" appeared on the screen, the sample was deemed negative for the COVID-19 virus antigen, with a measurement less than or equal to 150. If "E" appeared on the screen, the result was deemed invalid, requiring a retest using a new sample and device. The portable mini-potentiostat that was used in this study is a commercially available diagnostic device compliant with IVD Directive 98/79/EC (CE-marked; Declaration of Conformity issued April 26, 2022; Doc. No. DOC-01, Rev. 0, MARA Nanotech Korea Inc.)


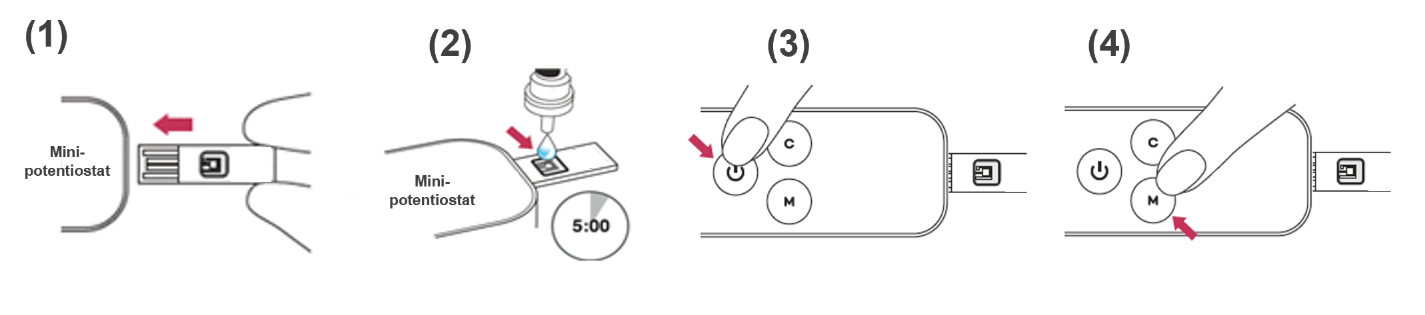
Figure S10. Illustration of the measurement process using a mini-potentiostat: (1) The sensor was inserted into the mini-potentiostat device. (2) One drop of the extracted sample was added onto the sensor and left at room temperature for 5 minutes. (3) The power button was pressed to turn on the mini-potentiostat, followed by pressing the measurement button to start the measurement. (4) The result was checked on the screen after 10 seconds.

Table S1. Correlation of viral load with C_t_ values for SARS-CoV-2 genes: Relationship between viral concentration (pfu/mL) and C_t_ values for RdRp, E, and N genes determined using the RT-PCR.

| Concentration (pfu/mL) | C_t_ value | | |
| --- | --- | --- | --- |
|  | RdRp gene | E gene | N gene |
| 1.0 × 10^3^ | 24.52 | 26.61 | 22.47 |
| 5.0 × 10^2^ | 25.63 | 27.88 | 23.68 |
| 2.5 × 10^2^ | 26.38 | 28.73 | 24.38 |
| 1.25 × 10^2^ | 28.07 | 30.68 | 26.11 |
| 6.25 × 10^1^ | 29.12 | 31.58 | 27.00 |
| 3.13 × 10^1^ | 30.17 | 32.49 | 28.01 |
| 1.56 × 10^1^ | 31.07 | 33.20 | 29.06 |

Table S2. RT-PCR confirmation of negative patient samples in LOD calculation: C_t_ values from RT-PCR assays verifying the absence of SARS-CoV-2 RNA in clinical samples used as negative matrices for spiking during LOD assessment.

| NO. | RdRp gene | E gene | N gene | IC Value |
| --- | --- | --- | --- | --- |
| 1 | Negative | Negative | Negative | 24.11 |
| 2 | Negative | Negative | Negative | 24.61 |
| 3 | Negative | Negative | Negative | 23.57 |

Table S3. RT-PCR confirmation of negative patient samples in variant virus experiment evaluation: C_t_ values from RT-PCR assays verifying the absence of SARS-CoV-2 RNA in clinical samples used as negative matrices for antigen spiking during SARS-CoV-2 variant detection experiments.

| No. | RdRp gene | E gene | N gene | IC Value |
| --- | --- | --- | --- | --- |
| 1 | Negative | Negative | Negative | 24.8 |
| 2 | Negative | Negative | Negative | 24.7 |
| 3 | Negative | Negative | Negative | 24.39 |

Table S4. Pathogen panel for cross-reactivity testing: Concentrations of tested pathogens (viruses and bacteria) used in the cross-reactivity assay, along with their respective manufacturers

| NO. | Organism | Abbreviation | Concentration | Manufacturer |
| --- | --- | --- | --- | --- |
| 1 | Human coronavirus 229E | HCoV-229E | 3.0×10^5^ (pfu/mL) | Korea University |
| 2 | Human coronavirus OC43 | HCoV-OC43 | 4.4×10^5^ (pfu/mL) | Korea University |
| 3 | Human Adenovirus 1 | HAdV-1 | 2.0×10^5^ (pfu/mL) | Korea University |
| 4 | Human metapneumovirus | HMPV | 1.0×10^5^ (pfu/mL) | Korea University |
| 5 | Human parainfluenza virus 1 | HPIV-1 | 1.8×10^5^ (pfu/mL) | Korea University |
| 6 | Human parainfluenza virus 2 | HPIV-2 | 1.0×10^5^ (pfu/mL) | Korea University |
| 7 | Human parainfluenza virus 3 | HPIV-3 | 1.5×10^5^ (pfu/mL) | Korea University |
| 8 | Human parainfluenza virus 4a | HIPV-4a | 1.15×10^5^ (pfu/mL) | Korea University |
| 9 | Human Influenza A virus (H3N2) | FLUAV | 1.0×10^5^ (pfu/mL) | Korea University |
| 10 | Human Influenza B virus | FLUBV | 1.2×10^5^ (pfu/mL) | Korea University |
| 11 | Human Enterovirus 70 | HEV70 | 1.1×10^5^ (pfu/mL) | Korea University |
| 12 | Human respiratory syncytial virus A | HRSV-A | 1.0×10^5^ (pfu/mL) | Korea University |
| 13 | Human rhinovirus 1B | HRV-1B | 1.2×10^5^ (pfu/mL) | Korea University |
| 14 | Human coronavirus NL63 | HCoV-NL63 | 1.0×10^5^ (pfu/mL) | BioNano Health Guard Research Center |
| 15 | Haemophiles parainfluenza | HPI | 1.0×10^5^ (pfu/mL) | BioNano Health Guard Research Center |
| 16 | Bordetella pertussis bacteria | - | 1.0×10^6^ (cfu/mL) | BioNano Health Guard Research Center |
| 17 | Mycoplasma pneumonia bacteria | - | 1.0×10^6^ (cfu/mL) | BioNano Health Guard Research Center |
| 18 | Legionella pneumophila bacteria | - | 1.0×10^6^ (cfu/mL) | BioNano Health Guard Research Center |
| 19 | Staphylococcus aureus bacteria | - | 1.0×10^6^ (cfu/mL) | BioNano Health Guard Research Center |
| 20 | Streptococcus pyogenes bacteria | - | 1.0×10^6^ (cfu/mL) | BioNano Health Guard Research Center |
| 21 | Staphylococcus epidermidis bacteria | - | 1.0×10^6^ (cfu/mL) | BioNano Health Guard Research Center |
| 22 | Candida albicans bacteria | - | 1.0×10^6^ (cfu/mL) | BioNano Health Guard Research Center |
| 23 | Chlamydia pneumonia bacteria | - | 1.0×10^6^ (cfu/mL) | BioNano Health Guard Research Center |
| 24 | Human Coronavirus HKU1 | HKU1 | 100 (ng/ml) | Sino Biological |
| 25 | MERS coronavirus (Recombinant) | MERS | 100 (ng/ml) | Sino Biological |
| 26 | SARS coronavirus (Recombinant) | SARS | 100 (ng/ml) | Sino Biological |

Table S5. RT-PCR confirmation of negative patient samples in cross-activity evaluation: C_t_ values from RT-PCR assays verifying the absence of SARS-CoV-2 RNA in clinical samples used as negative matrices for cross-activity experiments.

| No. | RdRp gene | E gene | N gene | IC Value |
| --- | --- | --- | --- | --- |
| 1 | Negative | Negative | Negative | 23.91 |
| 2 | Negative | Negative | Negative | 24.77 |
| 3 | Negative | Negative | Negative | 24.04 |

Table S6. RT-PCR confirmation of negative patient samples in media interference evaluation: C_t_ values from RT-PCR assays verifying the absence of SARS-CoV-2 RNA in clinical samples used as negative matrices for media matrix interference experiments.

| No. | RdRp gene | E gene | N gene | IC Value |
| --- | --- | --- | --- | --- |
| 1 | Negative | Negative | Negative | 24.75 |
| 2 | Negative | Negative | Negative | 24.51 |
| 3 | Negative | Negative | Negative | 24.86 |

Table S7. Tested interferents for nanowell biosensor specificity: Concentrations of potentially interfering substances tested for cross-reactivity with NW-Biosen.

| Interfering substances | Concentration | Interfering substances | Concentration |
| --- | --- | --- | --- |
| Whole Blood | 4% | Afrin (Oxymetazoline) | 15% v/v |
| Mucin | 0.50% | Nasal wash (Alkalol) | 1:10 dilution |
| Chloraseptic (Menthol/Benzocaine) | 1.5 mg/mL | Zicam | 5% v/v |
| Naso GEL (NeilMed) | 5% v/v | Sore Throat Phenol Spray | 15% v/v |
| CVS Nasal Drops (Phenylephrine) | 15% v/v | Tobramycin | 4 μg/mL |
| Mupirocin | 10 mg/mL | Tamiflu  (Oseltamivir Phosphate) | 5 mg/mL |
| Fluticasone Propionate | 5% v/v |  |  |

Table S8. Non-electrochemical techniques for detection of COVID-19 in clinical samples

| No | Technique | Detection method | Target biomarker | Sample type | Processing time | Clinical performance | Key features | Ref. |
| --- | --- | --- | --- | --- | --- | --- | --- | --- |
| 1 | RT-PCR (gold standard) | Reverse transcription qPCR | SARS-CoV-2 RNA | Swab/ 50 nasal samples | 2-6 h | Sensitivity: 72%;  specificity 98.1% | Standard of care; requires lab instrumentation | (1) |
| 2 | AI prediction model | Logistic regression (AI on blood counts | Complete blood count parameters | 2777 blood samples | ~30 min | Sensitivity: 85–93%; negative predictive value: 92–99% | Samples from 4 clinical sites in the US and South Korea | (2) |
| 3 | Deep learning-enhanced RT-LAMP | Isothermal amplification + fluorescence/turbidity | SARS-CoV-2 RNA | Swab, 250 saliva samples | 45 min | Sensitivity: 97.22%; specificity: 96.66% | Combined with deep learning system; portable | (3) |
| 4 | CRISPR-based | Cas12 collateral cleavage + fluorescence Lateral flow readout | SARS-CoV-2 RNA | Swab, 78 Oro-/nasopharyngeal samples | 30-40 min | 95% positive predictive; 100% negative predictive | Qualitative assay, LOD: 10 copies per µl input | (4) |
| 5 | Euroimmun ELISA kit | Plate-based immunoassay | Antibodies (IgG/IgM serology) | Serum, 167 samples | 2-3 h | Sensitivity >90% (≥14 days post onset),  <40% early infection;  specificity: ~95% | Good for past-infection (≥14 days post onset) | (5) |
| 6 | Optical immunoassay | Lateral flow immunochromatographic assay | SARS-CoV-2 antigen | Swab, 806 nasopharyngeal samples | ~15-20 min | Sensitivity: 85.4%; specificity: 99.7% | Tested for variants: Alpha, Beta, Delta, Gamma, Kappa, Omicron | (6) |
| 7 | Optical immunoassay (antibody fragments) | Surface plasmon field-enhanced fluorescence | Nucleocapsid (NP) antigen | Nasopharyngeal, nasal, saliva, 35 samples | ~20 min | Sensitivity: 100% (NP), 92% (nasal), and 62.5% (saliva); specificity 100% (NP),  90% (nasal), 100% (saliva) | LOD: 65.1 pg/mL (NP), 0.2 pg/mL (nasal), 1.5 pg/mL (saliva) | (7) |
| 8 | Magnetic immunoassay | Chemiluminescent enzyme immunoassay (Lumipulse) | NP antigen | Swab, 376 Oro-/nasopharyngeal samples | ~35 min | Sensitivity 96%,  specificity 98% | Fully automated; quantitative readout | (8) |
| 9 | Targeted proteomics | liquid chromatography–tandem mass spectrometry | two tryptic peptides from nucleocapsid | Swab, 985 Oro-/nasopharyngeal samples | 2.5 min per sample | Sensitivity: 83.6%,  specificity: 93.3% | High throughput, fully automated, LOD: 2.7 and 3.2 ng/mL for the two NP peptides | (9) |

Table S9. Clinical performance electrochemical platforms in detection of SARS-CoV-2

| No | Sensor structure | Detection method | Receptor | Target biomarker | Sample type | LOD | Processing time | Clinical performance | Key features | Ref. |
| --- | --- | --- | --- | --- | --- | --- | --- | --- | --- | --- |
| 1 | Flat planar (screen-printed electrode, SPE with carbon black | Differential pulse voltammetry (DPV) | Antibody | Spike (S) and nucleocapsid protein (N) | Untreated saliva | 19 ng/mL (S), 8 ng/mL (N) | 30 min | 24 samples | Magnetic bead-based immunoassay | (10) |
| 2 | Flat planar (magnetophoretic well/slit) | CA | Antibody | Nucleocapsid protein | Nasal swab | 8.89 ng/mL, 78.02 pfu/mL | 10 min | Sensitivity: 100%, specificity: 100% | Magnetophoretic electrochemical platform | (11) |
| 3 | Flat planar (porous, paper-based, porous) | SWV | Antibody | Anti-nucleocapsid IgG (antibody) | Serum | — | 4 min | 6 samples | Paper-based, nanoparticle-labeled, real-time monitoring | (12) |
| 4 | Flat planar (nano-roughened surface) | Resistance | Antigen | Spike-specific antibodies | Serum | ~100 pM | 30 min | 8 samples | High throughput, quantitative, enzymatically amplified metallization on nanostructured surfaces, 96 well plate | (13) |
| 5 | 3D porous metal-organic framework (MOF) | CV, DPV | Antibody | Protein (antigen) | Nasal swab | 60 fM | 20 min | 7 samples | Ag-MOF deposited on glassy carbon electrode | (14) |
| 6 | Flat (magnetic beads + SPE) | SWV | DNA probe | SARS-CoV-2 RNA and influenza A RNA | Saliva | SARS-CoV-2: 5 fmol L⁻¹; Influenza A: 1 fmol L⁻¹ | ~60 min | Sensitivity: 0.921 (SARS-CoV-2), 0.881 (influenza A) | Molecular discrimination between COVID-19 and Influenza | (15) |
| 7 | Flat (magnetic enhanced) | CA | Antibody | Spike protein | Nasopharyngeal swabs | 71.08 ng/mL | ~90 min | Sensitivity: 96.04%, specificity:  87.75% | Signal enhancement by magnetic beads; smartphone compatibility, Spike-ACE2 complex | (16) |
| 8 | Flat planar (molecularly imprinted polymer- coated electrode) | CV, SWV | MIP | Spike protein (S1) | Nasopharyngeal swab, PBS | 15 fM (PBS), 64 fM (clinical) | 20 min | 3 samples | Reusable polymer receptor; compatibility with portable potentiostat for POC testing | (17) |
| 9 | Flat planar | Current−voltage signal | SARS-CoV-2 ssDNA | nucleocapsid phosphoprotein (N-gene) | nasopharyngeal swab | 6.9 copies/μL | Real-time | 48 samples, sensitivity: 231 (copies μL^−1^) ^−1^ | Gold nanoparticle labeled | (18) |
| 10 | Flat planar | EIS | Multimeric aptamer | Spike protein (SP) | Saliva | < 2 fg/ml | 10 min | 13 samples | Nanostructured (NMIs) electrode | (19) |
| 11 | Flat planar | Single-frequency impedance measurement | Multimeric aptamer | Nucleocapsid protein | Saliva | 0.4 pg/mL | 18 min | 37 samples, sensitivity: 100%, specificity: 100% | Machine-learning-enhanced analysis, portable | (20) |
| 12 | Flat planar | CV, SWV | Aptamer and antibody | Protein and antibody | Saliva, nasal swabs | 7.62 fg/mL (S), 1 fg/mL (N) | 10 min | 5 samples | Multiplexed biochips (simultaneous detection); low-cost, one-step testing | (21) |
| 13 | Flat planar (miniaturized, graphene) | SWV, DPV | Self-actuated e-DNA | SARS-CoV-2 IVT RNA | Nasopharyngeal swab | ~2 copies/µL | ~30 min | 42 samples | Portable smartphone-based system, graphene microelectrode | (22) |
| 14 | Flat planar | EIS | Antibody | Nucleocapsid N protein | Saliva | 0.227 (glassy carbon), 0.334 (boron-doped diamond) and 0.362 ng/mL (gold) | <10 min | 10 samples | Includes molecular dynamics methods | (23) |
| 15 | Flat planar GCE modified with Zinc Oxide nanoflowers | CV, EIS, DPV | DNA probes | SARS-CoV-2 RNA | Clinical nasopharyngeal swabs in VIM (no extraction) | 14.137 fM (E gene; controls) | ~45 min | 48 samples,  sensitivity: 100%, specificity: 100% | Works directly in VIM | (24) |
| 16 | Nanowell array | CA | Antibody | Nucleocapsid protein | Anterior nasal swab | 60 pfu/mL | ~10 min | 492 samples, (prospective): sensitivity: 93.02%, specificity: 98.73%  (retrospective): sensitivity: 78.72%, specificity: 97.14% | Large-scale population testing; extensive cross-reactivity evaluation; smartphone-compatible POC, portable | This work |

Table S10. Nanostructured electrochemical biosensors evaluated using human-derived samples

| No | Nanostructure type | Sensor platform | Detection method | Target biomarker | Sample type | Clinical evaluation | LOD | Key features | Ref. |
| --- | --- | --- | --- | --- | --- | --- | --- | --- | --- |
| 1 | Nanowell array | Label-free impedance sensing chip | Non-faradaic EIS | Cortisol | Human serum, n = 65 | Correlation vs ELISA (R² = 0.91) | 0.5 μg/dL | Real-time detection; low volume (≤5 µL) | (25) |
| 2 | Nanowell array (wireless) | Wireless-powered impedance biosensor | EIS | Tumor Necrosis Factor-α (TNF-α) | Rheumatoid arthritis serum | RA vs healthy discrimination (p = 0.0089) | N/A | Remote readout capability | (26) |
| 3 | Nanowell multiplex array | Integrated impedance array | EIS | TNF-α, Interleukin-6 (IL-6), Interleukin-6 (IL-10) | Human serum (longitudinal study) | Agreement with Luminex assay | N/A | Multiplex nanoconfinement sensing | (27) |
| 4 | Gold nanodendrites (3D) | Au nanodendritic microelectrode | DPV | Cardiac troponin I | Human serum, n = 50 | ROC AUC = 0.97 vs ELISA | 0.21 pg/mL | High-surface-area signal amplification | (28) |
| 5 | Single-wall carbon nanotube (SWCNT) microwell array | SWCNT forest electrochemiluminescence (ECL) microwell chip | ECL | Prostate-specific antigen (PSA); IL-6 | Human serum, n = 6 | Agreement with ELISA (R ≈ 0.999) | PSA: 1 pg/mL; IL-6: 0.25 pg/mL | Multiplex nanoconfinement | (29) |
| 6 | Boron-doped diamond | Diamond microelectrode | CV, DPV | Sodium nitrite | Human urine, n=5 | Linear response in human urine (R² = 0.9914) | 0.82 mg/L | Enzyme-free; no added reagents | (30) |

**References**:

1. A. Garg, U. Ghoshal, S. S. Patel, D. V. Singh, A. K. Arya, S. Vasanth, A. Pandey, N. Srivastava, Evaluation of seven commercial RT-PCR kits for COVID-19 testing in pooled clinical specimens. *J. Med. Virol.* **93**, 2281–2286 (2021).

2. R. P. Joshi, V. Pejaver, N. E. Hammarlund, H. Sung, S. K. Lee, A. Furmanchuk, H.-Y. Lee, G. Scott, S. Gombar, N. Shah, S. Shen, A. Nassiri, D. Schneider, F. S. Ahmad, D. Liebovitz, A. Kho, S. Mooney, B. A. Pinsky, N. Banaei, A predictive tool for identification of SARS-CoV-2 PCR-negative emergency department patients using routine test results. *J. Clin. Virol.* **129**, 104502 (2020).

3. W. Waheed, S. Saylan, T. Hassan, H. Kannout, H. Alsafar, A. Alazzam, A deep learning-driven low-power, accurate, and portable platform for rapid detection of COVID-19 using reverse-transcription loop-mediated isothermal amplification. *Sci. Rep.* **12**, 4132 (2022).

4. J. P. Broughton, X. Deng, G. Yu, C. L. Fasching, V. Servellita, J. Singh, X. Miao, J. A. Streithorst, A. Granados, A. Sotomayor-Gonzalez, K. Zorn, A. Gopez, E. Hsu, W. Gu, S. Miller, C.-Y. Pan, H. Guevara, D. A. Wadford, J. S. Chen, C. Y. Chiu, CRISPR–Cas12-based detection of SARS-CoV-2. *Nat. Biotechnol.* **38**, 870–874 (2020).

5. J. Van Elslande, E. Houben, M. Depypere, A. Brackenier, S. Desmet, E. André, M. Van Ranst, K. Lagrou, P. Vermeersch, Diagnostic performance of seven rapid IgG/IgM antibody tests and the Euroimmun IgA/IgG ELISA in COVID-19 patients. *Clin. Microbiol. Infect.* **26**, 1082–1087 (2020).

6. S. A. Young, H. Zhang, J. Rodriguez, D. Mishkin, W. Paine, L. Seyfried, L. Hargrove, D. L. Broyles, C. Lindberg, A. Purushothaman, S. House, Clinical evaluation of the Healgen rapid COVID-19 antigen test as a point-of-care diagnostic tool. *Immun. Inflamm. Dis.* **13**, e70228 (2025).

7. N. Ashizawa, T. Takazono, K. Ota, Y. Ito, T. Hirayama, K. Takeda, S. Ide, N. Iwanaga, A. Fujita, M. Tashiro, N. Hosogaya, N. Akamatsu, K. Kosai, T. Tanaka, H. Kobayashi, R. Yamauchi, C. Segawa, H. Koizumi, N. Taka, H. Hishigaki, H. Mukae, Pre-clinical and clinical evaluation of a surface plasmon field-enhanced fluorescence spectroscopy (SPFS) antigen test for detecting SARS-CoV-2. *J. Infect. Chemother.* **31**, 102504 (2025).

8. A. Petruzziello, R. Sabatino, L. A. Catapane, C. De Falco, A. Petti, E. Tripaldelli, G. Loquercio, A. Annecchiarico, A. Salzillo, E. Caradonna, P. Maggi, Analytical performance evaluation of Lumipulse® SARS-CoV-2 antigen assay in 392 asymptomatic patients. *J. Clin. Lab. Anal.* **37**, e24867 (2023).

9. K. H. M. Cardozo, A. Lebkuchen, G. G. Okai, R. A. Schuch, L. G. Viana, A. N. Olive, C. dos Santos Lazari, A. M. Fraga, C. F. H. Granato, M. C. T. Pintão, V. M. Carvalho, Establishing a mass spectrometry-based system for rapid detection of SARS-CoV-2 in large clinical sample cohorts. *Nat. Commun.* **11**, 6201 (2020).

10. L. Fabiani, M. Saroglia, G. Galatà, R. De Santis, S. Fillo, V. Luca, G. Faggioni, N. D’Amore, E. Regalbuto, P. Salvatori, G. Terova, D. Moscone, F. Lista, F. Arduini, Magnetic beads combined with carbon black-based screen-printed electrodes for COVID-19: A reliable and miniaturized electrochemical immunosensor for SARS-CoV-2 detection in saliva. *Biosens. Bioelectron.* **171**, 112686 (2021).

11. N. Fukana, J. Park, G. J. Silva Junior, L. E. Malsick, E. N. Gallichotte, G. D. Ebel, B. J. Geiss, D. S. Dandy, M. Bertotti, D. Nacapricha, T. A. Baldo, C. S. Henry, Magnetophoretic slider assay for electrochemical detection of SARS-CoV-2 nucleocapsid protein in nasal swab samples. *Biosens. Bioelectron.* **271**, 117048 (2025).

12. L. Bezinge, A. J. deMello, C.-J. Shih, D. A. Richards, Quantitative reagent monitoring in paper-based electrochemical rapid diagnostic tests. *Lab Chip* **24**, 3651–3657 (2024).

13. H. Zhang, N. Rafat, J. Rudge, S. P. Peddireddy, Y. N. Kim, T. Khan, A. Sarkar, High throughput electronic detection of biomarkers using enzymatically amplified metallization on nanostructured surfaces. *Anal. Methods* **16**, 7854–7863 (2024).

14. S. Adel, A. Firoozbakhtian, H. Rabbani, M. Hosseini, A. B. Pebdeni, N. Sadeghi, J. Gilnezhad, M. R. Ganjali, COVID-19 electrochemical immunosensor with Ag-MOF: Rapid and high-selectivity nasal swab testing for effective detection. *Anal. Biochem.* **689**, 115500 (2024).

15. D. J. A. dos Santos, T. R. de Oliveira, G. M. de Araújo, H. Pott-Junior, M. E. Melendez, E. C. Sabino, O. D. Leite, R. C. Faria, An electrochemical genomagnetic assay for detection of SARS-CoV-2 and Influenza A viruses in saliva. *Biosens. Bioelectron.* **255**, 116210 (2024).

16. V. Vásquez, J. Orozco, Clinical validation of SARS-CoV-2 electrochemical immunosensor based on the spike-ACE2 complex. *J. Virol. Methods* **327**, 114940 (2024).

17. A. G. Ayankojo, R. Boroznjak, J. Reut, A. Öpik, V. Syritski, Molecularly imprinted polymer based electrochemical sensor for quantitative detection of SARS-CoV-2 spike protein. *Sens. Actuators B Chem.* **353**, 131160 (2022).

18. M. Alafeef, K. Dighe, P. Moitra, D. Pan, Rapid, ultrasensitive, and quantitative detection of SARS-CoV-2 using antisense oligonucleotides directed electrochemical biosensor chip. *ACS Nano* **14**, 17028–17045 (2020).

19. S. V. Hamidi, A. K. Jahromi, I. I. Hosseini, R. S. Moakhar, C. Collazos, Q. Pan, C. Liang, S. Mahshid, Surface-based multimeric aptamer generation and bio-functionalization for electrochemical biosensing applications. *Angew. Chem. Int. Ed.* **63**, e202402808 (2024).

20. P. Sen, Z. Zhang, S. Sakib, J. Gu, W. Li, B. R. Adhikari, A. Motsenayi, J. L’Heureux-Hache, J. C. Ang, G. Panesar, B. J. Salena, D. Yamamura, M. S. Miller, Y. Li, L. Soleymani, High-precision viral detection using electrochemical kinetic profiling of aptamer-antigen recognition in clinical samples and machine learning. *Angew. Chem. Int. Ed.* **63**, e202400413 (2024).

21. F. Jiang, Z. Xiao, T. Wang, J. Wang, L. Bie, L. Saleh, K. Frey, L. Zhang, J. Wang, Rapid and sensitive multiplex detection of COVID-19 antigens and antibody using electrochemical immunosensor-/aptasensor-enabled biochips. *Chem. Commun.* **58**, 7285–7288 (2022).

22. D. Ji, M. Guo, Y. Wu, W. Liu, S. Luo, X. Wang, H. Kang, Y. Chen, C. Dai, D. Kong, H. Ma, Y. Liu, D. Wei, Electrochemical detection of a few copies of unamplified SARS-CoV-2 nucleic acids by a self-actuated molecular system. *J. Am. Chem. Soc.* **144**, 13526–13537 (2022).

23. W. Białobrzeska, M. Ficek, B. Dec, S. Osella, B. Trzaskowski, A. Jaramillo-Botero, M. Pierpaoli, M. Rycewicz, Y. Dashkevich, T. Łęga, N. Malinowska, Z. Cebula, D. Bigus, D. Firganek, E. Biega, K. Dziąbowska, M. Brodowski, M. Kowalski, M. Panasiuk, B. Gromadzka, R. Bogdanowicz, Performance of electrochemical immunoassays for clinical diagnostics of SARS-CoV-2 based on selective nucleocapsid N protein detection: Boron-doped diamond, gold and glassy carbon evaluation. *Biosens. Bioelectron.* **209**, 114222 (2022).

24. S. Alafeef, K. Dighe, P. Moitra, D. Pan, ZnO nanoflower-based electrochemical platform for direct SARS-CoV-2 RNA detection in clinical swab samples. *ACS Appl. Nano Mater.* 7, 1234–1245 (2024).

25. S. R. Mahmoodi, P. Xie, D. P. Zachs, E. J. Peterson, R. S. Graham, C. R. W. Kaiser, H. H. Lim, M. G. Allen, M. Javanmard, Single-step label-free nanowell immunoassay accurately quantifies serum stress hormones within minutes. *Sci. Adv.* **7**, eabf4401 (2021).

26. Raji, H., et al., Wireless power-up and readout from a label-free biosensor. *Biomedical Microdevices* **27**, 2 (2025).

27. P. Xie, N. Song, W. Shen, M. Allen, M. Javanmard, “Nanowell Array Impedance Sensor for Label‐Free Quantification of Cytokines in Serum at Femtomolar Detection Limits,” in Proceedings of the 21st International Conference on Miniaturized Systems for Chemistry and Life Sciences (MicroTAS) (Chemical and Biological Microsystems Society, 2020).

28. Cen, S.-Y., et al., Label-free electrochemical immunosensor for ultrasensitive determination of cardiac troponin I based on porous fluffy-like AuPtPd trimetallic alloyed nanodendrites. *Microchemical Journal* **169**, 106568 (2021).

29. Sardesai, N. P., J. C. Barron, and J. F. Rusling, Carbon nanotube microwell array for sensitive electrochemiluminescent detection of cancer biomarker proteins. *Analytical Chemistry* **83**, 6698–6703 (2011).

30. Zhang, Z., et al., Electrochemical diagnosis of urinary tract infection using boron-doped diamond electrodes. *ACS Sensors* **8**, 4245–4252 (2023).
